# Bacillus subtilis Stressosome Sensor Protein Sequences Govern the Ability To Distinguish among Environmental Stressors and Elicit Different σ^B^ Response Profiles

**DOI:** 10.1128/mbio.02001-22

**Published:** 2022-11-21

**Authors:** Christopher W. Hamm, Daxton R. Butler, Matthew T. Cabeen

**Affiliations:** a Department of Microbiology and Molecular Genetics, Oklahoma State University, Stillwater, Oklahoma, USA; Max Planck Unit for the Science of Pathogens; University of Würzburg

**Keywords:** microfluidics, sigma factors, signal transduction, stress proteins, stress response

## Abstract

Bacteria use a variety of systems to sense stress and mount an appropriate response to ensure fitness and survival. Bacillus subtilis uses stressosomes—cytoplasmic multiprotein complexes—to sense environmental stressors and enact the general stress response by activating the alternative sigma factor σ^B^. Each stressosome includes 40 RsbR proteins, representing four paralogous (RsbRA, RsbRB, RsbRC, and RsbRD) putative stress sensors. Population-level analyses suggested that the RsbR paralogs are largely redundant, while our prior work using microfluidics-coupled fluorescence microscopy uncovered differences among the RsbR paralogs’ σ^B^ response profiles with respect to timing and intensity when facing an identical stressor. Here, we use a similar approach to address the question of whether the σ^B^ responses mediated by each paralog differ in the presence of different environmental stressors: can they distinguish among stressors? Wild-type cells (with all four paralogs) and RsbRA-only cells activate σ^B^ with characteristic transient response timing irrespective of stressor but show various response magnitudes. However, cells with other individual RsbR paralogs show distinct timing and magnitude in their responses to ethanol, salt, oxidative, and acid stress, implying that RsbR proteins can distinguish among stressors. Experiments with hybrid fusion proteins comprising the N-terminal half of one paralog and the C-terminal half of another argue that the N-terminal identity influences response magnitude and that determinants in both halves of RsbRA are important for its stereotypical transient σ^B^ response timing.

## INTRODUCTION

Bacteria must be able to sense and respond appropriately to stressful environments to survive and grow. Bacterial responses to stress can be mediated by many different mechanisms that connect environmental sensing to gene expression, but one common mechanism employs alternative sigma factors, such as the σ^S^ general stress response, the σ^38^ stationary-phase and osmotic shock factor, or the σ^32^ heat shock factor of Escherichia coli (1–4). In Bacillus subtilis, as well as other related Gram-positive organisms, the general stress response (GSR) is mediated by σ^B^, which activates hundreds of genes (5–10). σ^B^ activates gene expression in response to energy depletion and during the stationary phase of growth, and it responds to a range of environmental stressors, such as high and low temperatures, salt, ethanol, pH changes, and oxidative and osmotic stress (7, 11–15). The characteristic σ^B^ transcriptional response to environmental stress in wild-type cells is transient: fast-onset, detectable in minutes, peaking within approximately 30 min, and subsiding thereafter (16–19).

To ensure a fast response, alternative sigma factors are present within cells but prevented from binding RNA polymerase until activating conditions are met. Under unstressed conditions, σ^B^ is held inactive by the anti-sigma factor RsbW (20). Activation is achieved when the anti-anti-sigma factor RsbV is dephosphorylated and preferentially bound by RsbW, freeing σ^B^ (21). RsbV can be dephosphorylated by either of two analogous proteins in response to starvation or environmental stressors. The energy stress response is mediated by RsbQ, which activates the RsbV-phosphatase RsbP; meanwhile, the environmental stress pathway is mediated by RsbT, which activates the RsbV-phosphatase RsbU (13, 14, 19, 22). While the pathway between RsbP or RsbU and the activation of σ^B^ is well characterized, the initial steps of stress sensing and transduction are less well understood. RsbU requires activation through binding to RsbT, which is typically sequestered by RsbR proteins and RsbS in a protein complex known as the stressosome (9, 18, 23). When stress is sensed by the stressosome, RsbT, which also has kinase activity, triggers its own release in association with phosphorylation of RsbR at Thr residues 171 and 205 and RsbS at Ser residue 59 (24). Mutation of the phosphorylated Ser residue to Ala in RsbS or mutational inactivation of RsbT kinase activity abrogates σ^B^ activation in Listeria monocytogenes (25) and B. subtilis (18, 26). Released RsbT activates RsbU, resulting in σ^B^ release from RsbW and subsequent activation of the GSR. The system is reset by RsbX, a phosphatase that dephosphorylates RsbR and RsbS, presumably allowing the stressosome to recapture RsbT and discontinuing activation of σ^B^ (27–29). The signal for RsbT to phosphorylate RsbR and RsbS and trigger its release from the stressosome is thought to be mediated by stress sensing by RsbR via a process that remains unknown.

**FIG 1:**
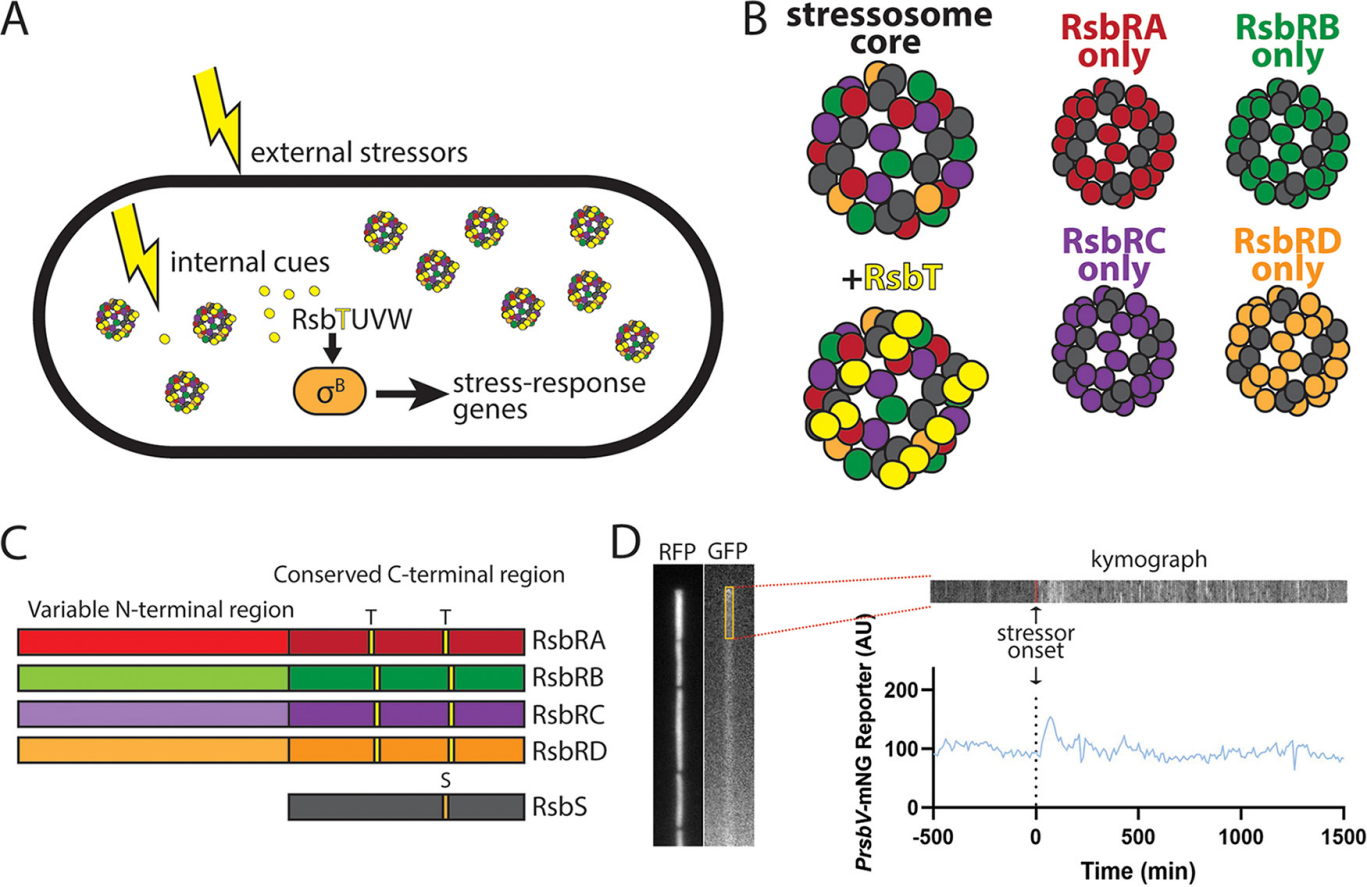
(A) Schematic of Bacillus subtilis cell containing stressosomes within the cytoplasm sensing stress and triggering the activation of σ^B^. Once activated, σ^B^ turns on gene expression of the general stress response, including the P*_rsbV_*-mNeonGreen reporter that we used to detect when σ^B^ is active. (B) Schematics of the stressosome complex in the wild type and stressosomes formed by single RsbR paralogs. Stressosomes are comprised of 20 RsbS proteins (gray) and 20 RsbR dimers (RsbRA in red, RsbRB in green, RsbRC in purple, and RsbRD in orange). All strains in this study contained the Δ*ytvA* deletion of the blue light sensor to prevent activation by fluorescence imaging. (C) Diagram of RsbR paralogs compared to one another, as well as RsbS, with their phosphorylation sites highlighted. RsbR paralogs and RsbS share a conserved C-terminal sulfate transporter and anti-sigma factor antagonist (STAS) domain, which makes up the core of the stressosome. (D) Image of a single cell lineage in a channel in the microfluidic device. The red fluorescent protein (RFP) image shows the cell boundaries, whereas the green fluorescent protein (GFP) image reports on σ^B^ activity. The yellow box is a 5 × 50-pixel region of interest (ROI) drawn in ImageJ for analysis, from which we obtained a kymograph depicting that ROI over time. The ROI was also used to obtain the mean GFP fluorescence value to plot over time for graph generation. Data curation and analysis were manually performed for each individual cell lineage.

Relatively few species appear to use stressosome complexes to respond to stress. The stressosome is best known in B. subtilis and Listeria monocytogenes, but similar complexes have been discovered in Vibrio vulnificus and Moorella thermoacetica (25, 30, 31). In B. subtilis, the stressosome complex, which is composed of RsbR and RsbS proteins and can additionally sequester RsbT, is responsible for sensing and responding to environmental stress by activating σ^B^ as described above (Fig. 1A) (18, 32). The stressosome itself is a large, 1.8-megadalton pseudoicosahedral structure with a core comprising 20 RsbR dimers, each bound to an RsbS protein (Fig. 1B) (17, 32). The RsbRS core can sequester up to 20 RsbT proteins (Fig. 1B) that are released upon sensing of stress (9, 33). There are thought to be 10 to 20 of these large stressosome complexes within the cytoplasm of each B. subtilis cell (Fig. 1A) (32). The stressosome appears to be the sensory component of the σ^B^ activation pathway, yet how it senses stress remains a mystery.

The N-terminal half of RsbR is thought to be a sensing domain, as it extends out into the cytoplasm as a “turret” from the stressosome core (32). Wild-type cells encode four RsbR paralogs, termed RsbRA, RsbRB, RsbRC, and RsbRD (Fig. 1C), that are thought to be present in the stressosome and whose genes are scattered around the chromosome. A fifth paralog known as YtvA is known to sense blue light using a light-oxygen-voltage (LOV) domain in its N-terminal half, but YtvA is unable to form stressosomes on its own *in vivo*, instead forming a heterotetramer with an RsbRA dimer (34–37). RsbRA, RsbRB, RsbRC, RsbRD, and YtvA share a conserved C-terminal sulfate transporter and anti-sigma factor antagonist (STAS) region with RsbS and have a non-heme globin variable N-terminal region thought to be the sensing domain (30, 32, 38). The presence of the sensory LOV domain in the N-terminal half of YtvA bolsters the idea that the N-terminal halves of the other RsbR paralogs also serve sensory functions. Within wild-type cells, the levels of RsbRA, RsbRB, and RsbRC appear to be comparable, while RsbRD is present at very low levels, rising only to 30% of the level of RsbRA during stressful conditions (38). It is possible to delete all but one paralog, and stressosomes comprised of only one type of RsbR protein will form (Fig. 1B), allowing study of individual RsbRs (16, 38, 39). Even stressosomes comprised of only RsbRD, which is present at 20% of the levels of the other single-RsbR proteins, are still able to prevent σ^B^ activation in the absence of stress (38), suggesting that all available RsbT is sequestered and further implying that wild-type cells harbor excess RsbT-seqestering capacity. B. subtilis strains containing single-RsbR paralogs maintain a σ^B^ response to environmental stressors (18), but our previous work using microfluidics coupled to fluorescence microscopy showed that individual RsbR paralogs each show a distinct σ^B^ response profile when subjected to the same ethanol stressor (16).

A long-standing question in the field asks why species like B. subtilis and L. monocytogenes encode multiple RsbR paralogs. Given that the GSR responds to a diversity of environmental stressors, one attractive hypothesis is that having multiple RsbRs broadens the sensory capacity of the stressosome, with different paralogs “specializing” in sensing different types of stress. Supporting this idea, in L. monocytogenes, the stressosome is used for sensing both environmental and nutritional stress, and in B. subtilis, RsbRC and RsbRD can sense nutritional stress in the absence of RsbRA and RsbRB (39). On the other hand, the demonstrated ability of single-RsbR strains to respond to a given stress (16, 18) raise the nonexclusive possibility that RsbRs respond to a common intracellular signal triggered by multiple environmental stressors. If all the RsbR paralogs respond to a common signal, then each paralog may merely modulate the strength and timing of the response rather than distinguishing among different stressors.

We previously examined the responses of wild-type and single-RsbR strains to a single environmental stressor (ethanol) using microfluidics coupled to fluorescence microscopy. That experimental approach allowed the visualization of single-cell σ^B^ responses to introduced stressors over long periods of time under uniform conditions (16, 40). We revealed previously unappreciated differences in the σ^B^ response profiles of individual RsbR paralogs, with each paralog showing different response dynamics (16). However, it has been unknown whether wild-type or single-RsbR strains can distinguish among different environmental stressors by enacting different σ^B^ response profiles. It is also unknown whether the response profile of a particular RsbR paralog is governed by its N-terminal variable region, its C-terminal conserved region, or a combination of both.

Here, we extended our microfluidics-based approach to investigate whether and how wild-type and single-RsbR *B. subtills* strains modulate their σ^B^ responses to different stressors. We challenged cells with a panel of four stressors: ethanol, sodium chloride, hydrogen peroxide, and mild acidification. We first validated our experimental approach by comparison with our previous results (16). We then found that whereas wild-type and RsbRA-only cells vary only in the magnitude of their response to different stressors, showing a characteristic transient response, other single-RsbR strains vary their responses to different stressors not only in magnitude but also in timing (shape of the response), suggesting that RsbR paralogs can indeed distinguish among different stressors. We also use a set of strains bearing RsbR hybrid fusion proteins to probe the effects of different N- and C-terminal regions on the σ^B^ response profile enacted by a particular RsbR sequence. Our results suggest that the N-terminal sequence exerts an effect on the magnitude of the response and that the characteristically transient response of RsbRA (which mimics the wild-type response) requires both halves of RsbRA.

## RESULTS

### Consistency between analytical pipelines for microfluidics-coupled fluorescence microscopy.

A key approach in our previous work (16) permitting us to detect subtle differences among the RsbR paralogs was microfluidics-coupled fluorescence microscopy. Unlike in bulk studies, the microfluidic environment in which cells are growing is both uniform and controllable, and σ^B^ responses are observed in single cells, allowing us to distinguish among different single-cell behaviors that might give rise to similar ensemble behaviors. However, as the present study was performed using different microfluidic devices, different imaging equipment, and a different analysis method in a different laboratory, we first sought to ensure that data from our present setup was comparable to our previously published data. As we had done previously, we used a P*_rsbV_*-mNeonGreen fluorescent reporter to detect σ^B^-directed GSR activity. We equilibrated cells in the microfluidic device under constant flow in the absence of stressor while imaging every 10 min for >500 min prior to adding a stressor. After switching cells to stressor-containing medium, we continued growing cells under constant stressed conditions while imaging at 10-min intervals for >1,500 min. Fluorescence quantification was performed using manual curation in ImageJ by drawing an region of interest (ROI) slightly smaller than the size of an individual cell and measuring the average pixel value within the ROI (Fig. 1D). We then subtracted the average background value from several measurements of unoccupied areas of the device and plotted fluorescence over time (Fig. 1D) to obtain individual cell traces.

**FIG 2:**
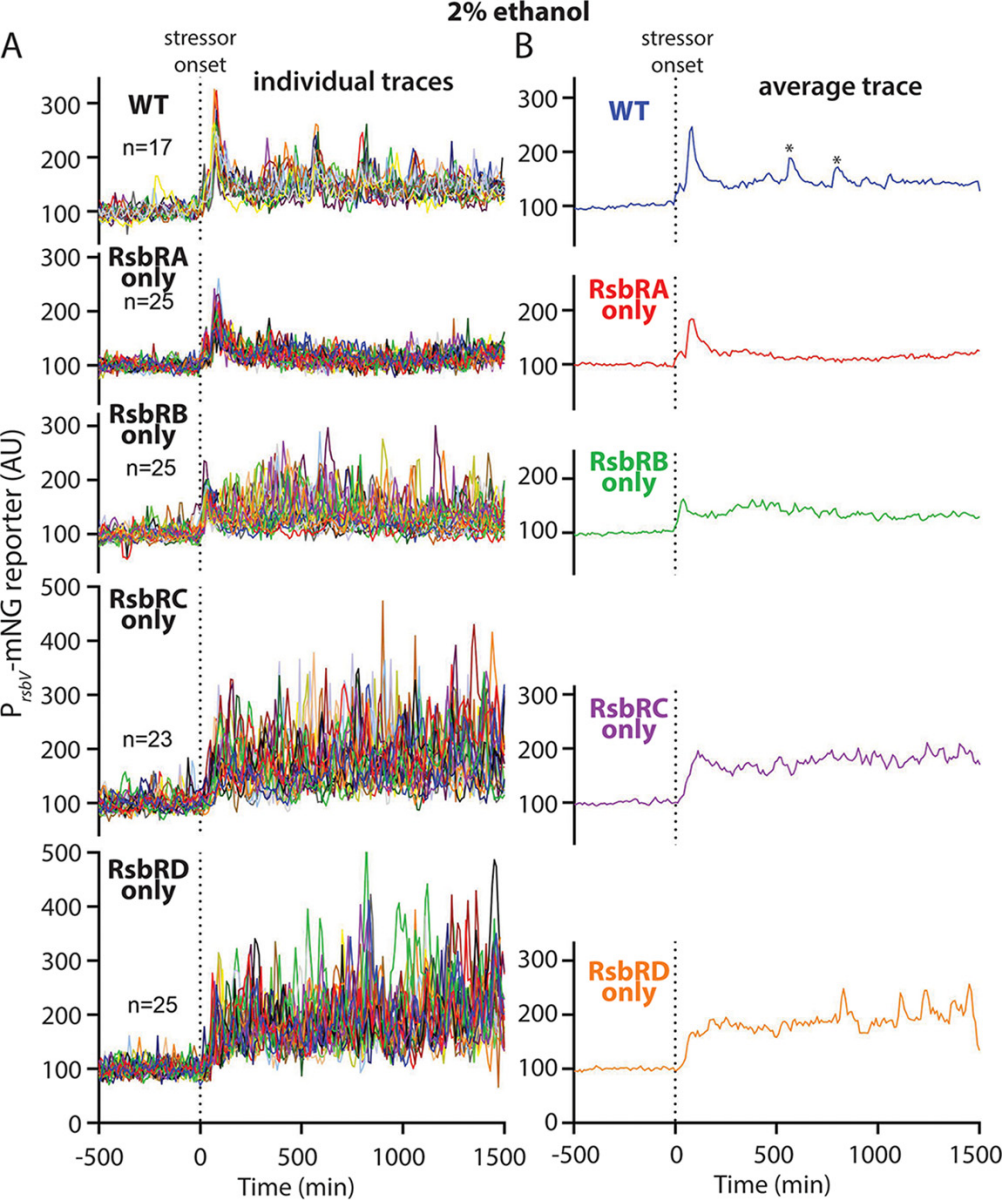
(A) Individual-lineage intensity traces of a stress-responsive P*_rsbV_*-mNeonGreen reporter in a wild-type (WT) strain (MTC1801) and strains containing only RsbRA (MTC1761), RsbRB (MTC1763), RsbRC (MTC1765), and RsbRD (MTC1767) before and after the addition (indicated by vertical dotted line) of 2% ethanol. (B) Average intensity of the P*_rsbV_*-mNeonGreen reporter corresponding to the single-cell traces from panel A. Asterisks in the EtOH trace denote peaks that were present in only some lineages corresponding to the same experimental replicate (see also Fig. S1) and hence are considered artifactual.

We compared our present imaging and analysis workflow to our previous work by using 2% ethanol as a stressor and assessing a wild-type strain and strains containing only RsbRA, RsbRB, RsbRC, or RsbRD. We observed a transient, synchronous response by wild-type cells that was very similar to the response we previously observed (Fig. 2A and 2B) (16). Cells containing only RsbRA exhibited a similar response to wild-type cells, while cells containing only RsbRC showed a slower-onset repeated response that was stochastic across individual cells, again matching our previous work (Fig. 2A and 2B). Again, consistent with our previous observations, RsbRB-only cells showed a RsbRC-like response but weaker in magnitude (Fig. 2A and 2B). We did notice a modest difference from our previous results (16) in the RsbRD-only response, which here lacked an initial response spike before an RsbRC-like repeated response (Fig. 2). After repeating this experiment several times, we are convinced of the accuracy of the result presented here. We conclude that our present experimental approach yields comparable results to our previous work despite minor technical and methodological differences, demonstrating the utility and consistency of the approach.

**FIG 3:**
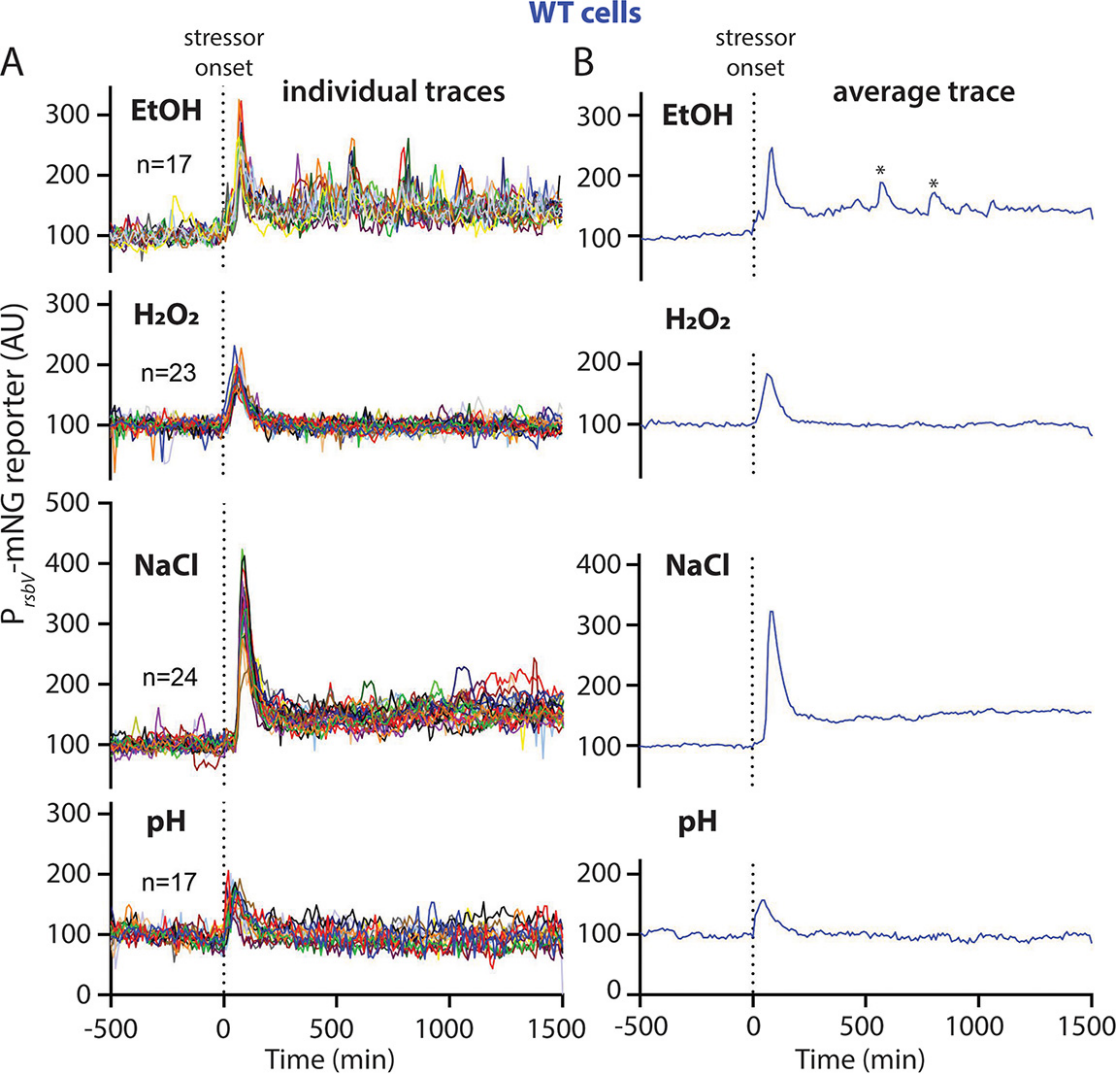
(A) Individual-lineage intensity traces of a stress-responsive P*_rsbV_*-mNeonGreen reporter in a wild-type strain (MTC1801) before and after the addition (indicated by vertical dotted line) of the indicated stressors. The stressors were 2% ethanol, 0.005% H_2_O_2_, 500 mM NaCl, and pH shift from 6.5 to 6.25. (B) Average intensity of the P*_rsbV_*-mNeonGreen reporter corresponding to the single-cell traces from panel A. Asterisks in the EtOH trace denote peaks that were present in only some lineages corresponding to the same experimental replicate (see also Fig. S1) and hence are considered artifactual.

10.1128/mbio.02001-22.1 FIG S1 P*_rsbV_-*mNeonGreen (σ^B^ reporter) traces for individual wild-type cell lineages in different stressors. Traces from individual cells, corresponding to the ensemble and average traces shown in Fig. 3, are shown. Each column shows traces from five individual cells challenged with the stressor shown at the top of the column. EtOH, 2% ethanol; H_2_O_2_, 0.005% hydrogen peroxide; NaCl, 500 mM NaCl; pH, shift from pH 6.5 to 6.25. Download FIG S1, JPG file, 1.8 MB. Copyright © 2022 Hamm et al. 2022 Hamm et al. https://creativecommons.org/licenses/by/4.0/ This content is distributed under the terms of the Creative Commons Attribution 4.0 International license.

### The wild-type σ^B^ response to four environmental stressors differs in magnitude but not timing.

We next approached the longstanding question of whether having multiple RsbR paralogs abets cells’ ability to sense a range of environmental stressors. Do cells with wild-type stressosomes (i.e., containing all four RsbR paralogs) differentiate among different stressors by altering the timing or magnitude of their σ^B^ responses? To answer this question, we assembled a panel of four well-known environmental stressors: ethanol, salt (NaCl), pH, and oxidative stress (thermal stress is also a well-known environmental stressor, but we limited our analysis to stressors that we could add to the medium) (7, 8, 11, 25, 31, 41–45). Previous studies analyzing the wild-type B. subtilis response to different environmental stressors showed the typical transient response irrespective of stressor (8, 11, 16, 46). However, as these studies were performed in bulk cultures, typically using β-galactosidase assays to assess the σ^B^ response at the population level, we hypothesized that our microfluidic approach might detect subtle differences among the responses to different stressors that manifest at the single-cell level.

One experimental design consideration was that microfluidic growth can change the tolerance of cells to stressors relative to batch growth. When a stressor is added to a closed culture, the concentration of that stressor may decline as it reacts with cell components, is metabolized, or is otherwise detoxified by the bacterial cells. In contrast, in a microfluidic device, fresh medium with a set concentration of stressor is continuously replenished, so that the stressor concentration never appreciably declines. We attribute to this difference our informal observations that stressors or antibiotics are lethal at lower concentrations in the microfluidic platform than in bulk culture (16). Hence, we found that we often had to lower the concentration or magnitude of a stressor relative to previous reports. Our initial tests showed that previously reported stressors, such as pH shift from 6.5 to 5.5 or challenge with 1 M NaCl as previously used (7, 11, 41), caused widespread cell lysis or death, with very few survivors that continued to grow and divide at a normal rate under the stress condition. Hence, we used empirically determined sublethal concentrations of stressors that stimulated the σ^B^ response without broadly killing cells. Guided by the literature and pilot studies, we used 2% ethanol, 500 mM NaCl, 0.005% H_2_O_2_, and acid stress from pH 6.5 to 6.25 as our stressors. We challenged wild-type cells with each stressor and used our current analysis method to assess their σ^B^ response profiles.

Wild-type cells always responded with the same characteristic σ^B^ response profile, irrespective of which stressor we used (Fig. 3; Fig. S1 and S6; Movies S1 to S4 at https://doi.org/10.5061/dryad.2ngf1vhs1). Individual cells synchronously activated σ^B^ upon introduction of the stressor (Fig. S1; Fig. S1 to S5 show separate graphs for single cells of each strain and condition), and the response was transient, peaking roughly 30 min after exposure and then declining toward the baseline nonactivated state despite continued exposure to the stressor (Fig. 3). However, we observed stressor-specific differences in the response magnitude. Acid and oxidative stress elicited a relatively weak response compared to ethanol and NaCl, with NaCl eliciting the strongest response (Fig. 3B). Moreover, only ethanol- and NaCl-challenged cells showed a weak but prolonged response after the initial response peak, not quite returning to the prestress baseline (Fig. 3B). Previous research has shown that the magnitude of the response can be affected by the concentration of the stressor used (7, 16, 46, 47). Because environmental stressors may stress cells in different ways, stressor concentrations are not directly comparable. Our initial testing suggested that at least for some stressors, H_2_O_2_ for instance, greater stressor concentrations cause cell death rather than eliciting a stronger σ^B^ response. However, none of the stressor concentrations used in this work substantially affected cell growth, arguing in favor of the stressors having a comparable effect on cells. Collectively, our results suggest that cells with wild-type stressosomes display a stereotypical transient and synchronous σ^B^ response pattern irrespective of stressor identity but that different stressors provoke different response magnitudes.

10.1128/mbio.02001-22.6 FIG S6 Average σ^B^ reporter traces grouped by stressor. For convenient comparison, the average traces for each strain (wild-type and each RsbR paralog on its own) in the indicated stressor are shown in different colors on the same plot. The left column shows the average traces with a shaded standard deviation envelope. The right column shows the same average traces without the envelope, for clarity. EtOH, 2% ethanol; H_2_O_2_, 0.005% hydrogen peroxide; NaCl, 500 mM NaCl; pH, shift from pH 6.5 to 6.25. Download FIG S6, JPG file, 4.0 MB. Copyright © 2022 Hamm et al. 2022 Hamm et al. https://creativecommons.org/licenses/by/4.0/ This content is distributed under the terms of the Creative Commons Attribution 4.0 International license.

### The RsbRA-only response closely resembles the wild-type response.

Our experimental approach, here (Fig. 2) and in previous work (16), revealed distinct σ^B^ response profiles for cells bearing stressosomes containing each individual RsbR paralog in isolation. Hence, we asked whether the σ^B^ response profiles of such single-RsbR strains change depending on stressor. We began by examining RsbRA-only cells, whose response profile closely matched the wild-type profile in our present work (Fig. 2) and in previous research, suggesting that RsbRA dominates the response in wild-type cells (16). We thus hypothesized that the RsbRA-only response might closely match the wild-type response across our panel of four stressors.

**FIG 4:**
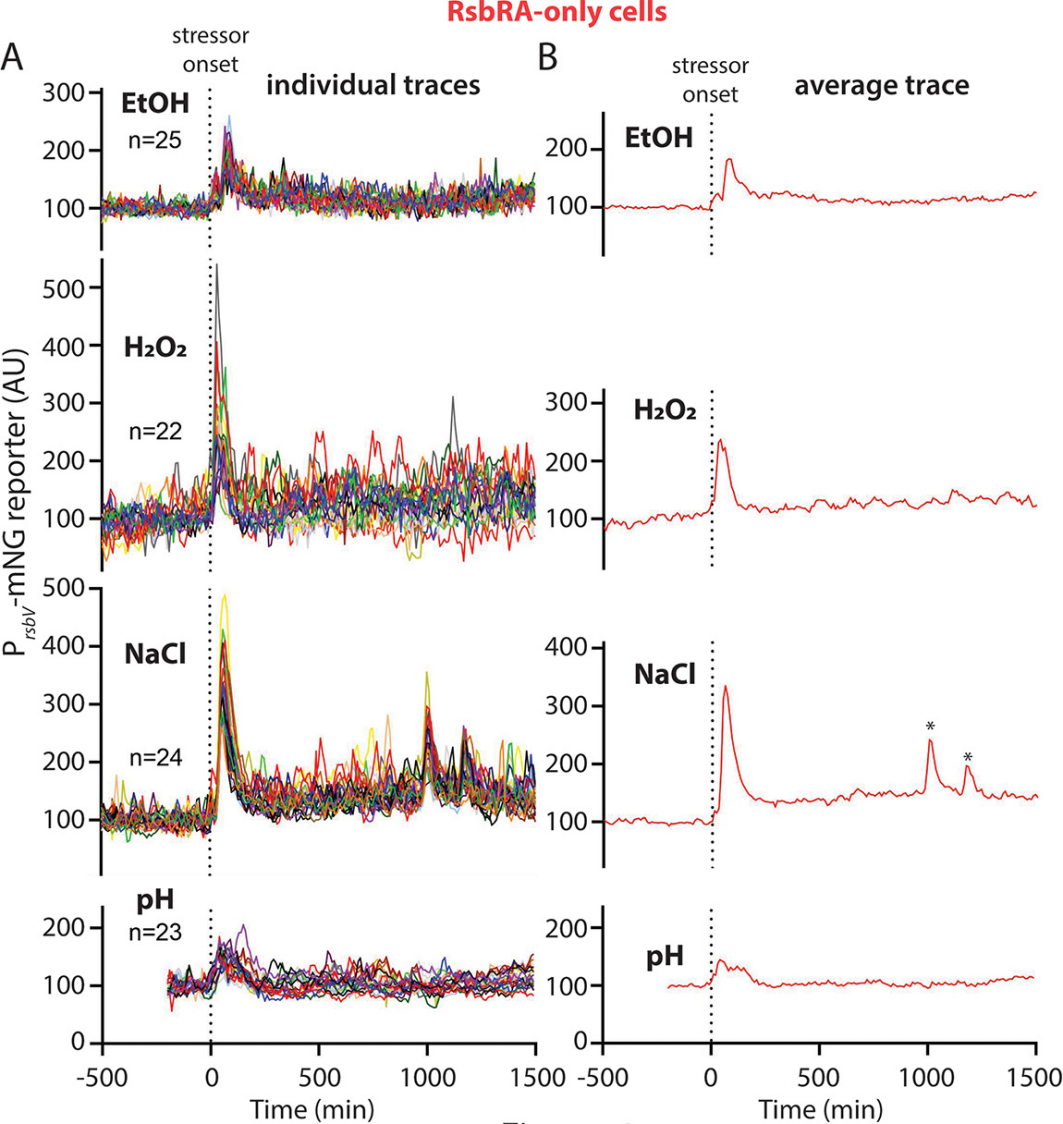
(A) Individual-lineage intensity traces of a stress-responsive P*_rsbV_*-mNeonGreen reporter in an RsbRA-only strain (MTC1761) before and after the addition (indicated by vertical dotted line) of the indicated stressors. The stressors were 2% ethanol, 0.005% H_2_O_2_, 500 mM NaCl, and pH shift from 6.5 to 6.25. (B) Average intensity of the P*_rsbV_*-mNeonGreen reporter corresponding to the single-cell traces from panel A. Asterisks in the NaCl trace denote peaks that were present in only some lineages corresponding to the same experimental replicate (see also Fig. S2) and hence are considered artifactual.

Indeed, our results revealed that the RsbRA-only response pattern largely resembles the wild type (Fig. 4; Fig. S2 and S6; Movies S5 to S8 at https://doi.org/10.5061/dryad.2ngf1vhs1). We observed the same stereotypical synchronous, transient response regardless of stressor identity, and the response magnitudes were also similar to the wild type (compare Fig. 3 and 4; Fig. S6). As in the wild type, NaCl stress provoked the strongest response, whereas pH induced the weakest response (Fig. 4B and 3B). One modest difference between wild-type and RsbRA-only cells was that H_2_O_2_ stress elicited a stronger response in the RA-only cells, with some individual cells peaking more than twice as high as in the wild type (Fig. 3A and 4A). Meanwhile, the response to 2% ethanol was slightly weaker in RsbRA-only cells. These data continue to support a model in which the RsbRA-type response largely dominates in wild-type cells containing all four RsbR paralogs. They also suggest the possibility that RsbRA is particularly sensitive to oxidative stress and that the presence of other RsbR paralogs has a moderating influence on the σ^B^ response to hydrogen peroxide.

10.1128/mbio.02001-22.2 FIG S2 P*_rsbV_-*mNeonGreen (σ^B^ reporter) traces for individual RsbRA-only cell lineages in different stressors. Traces from individual cells, corresponding to the ensemble and average traces shown in Fig. 4, are shown. Each column shows traces from five individual cells challenged with the stressor shown at the top of the column. EtOH, 2% ethanol; H_2_O_2_, 0.005% hydrogen peroxide; NaCl, 500 mM NaCl; pH, shift from pH 6.5 to 6.25. Download FIG S2, JPG file, 1.9 MB. Copyright © 2022 Hamm et al. 2022 Hamm et al. https://creativecommons.org/licenses/by/4.0/ This content is distributed under the terms of the Creative Commons Attribution 4.0 International license.

### The RsbRB-only response differs according to stressor and is insensitive to oxidative stress.

We next expanded our analysis to the remaining RsbR paralogs. Unlike wild-type and RsbRA-only cells, with their characteristically synchronous and transient response, our present (Fig. 2) and previous work (16) shows that ethanol stress elicits, to various degrees, repeated activation of σ^B^ in cells bearing one of the other RsbR paralogs. For example, in ethanol, RsbRB-only cells show a repeated response that is dissimilar to the pattern generated by RsbRA-only cells (Fig. 2A and 2B). While RsbRA-only cells responded comparably to different stressors, the responses differing mainly by magnitude, we asked whether cells bearing other RsbR paralogs would modulate their response timing based on stressor identity.

**FIG 5:**
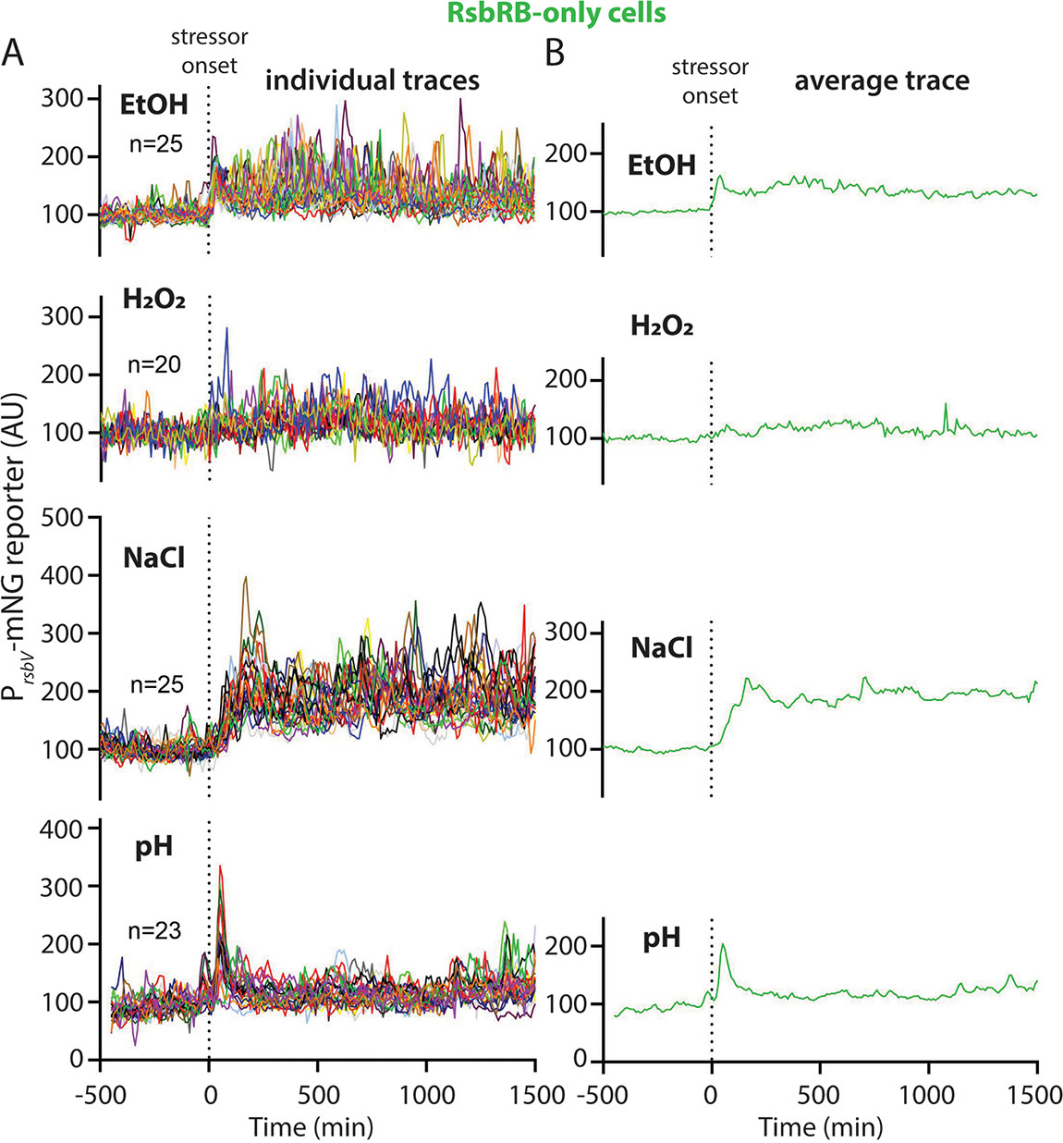
(A) Individual-lineage intensity traces of a stress-responsive P*_rsbV_*-mNeonGreen reporter in an RsbRB-only strain (MTC1763) before and after the addition (indicated by vertical dotted line) of the indicated stressors. The stressors were 2% ethanol, 0.005% H_2_O_2_, 500 mM NaCl, and pH shift from 6.5 to 6.25. (B) Average intensity of the P*_rsbV_*-mNeonGreen reporter corresponding to the single-cell traces from panel A. Representative individual traces are shown in Fig. S3.

Interestingly, we observed that RsbRB-only cells distinguish among different stressors with respect to the timing of the subsequent σ^B^ response patterns. In ethanol, as in our previous work (16), we observed a sustained response (Fig. 5B) manifesting at the single-cell level as stochastic, repeated response peaks (Fig. 5A; Movie S9 at https://doi.org/10.5061/dryad.2ngf1vhs1). We saw a qualitatively similar but stronger response in NaCl stress (Fig. 5A and 5B; Fig. S3 and S6; Movie S11 at https://doi.org/10.5061/dryad.2ngf1vhs1), with a sustained response that set RsbRB apart from the RsbRA and wild-type responses. Oxidative stress elicited a very weak, barely detectible repeated response compared to RsbRA-only or the wild type (compare Fig. 3B, 4B, and 5B; Movie S10 at https://doi.org/10.5061/dryad.2ngf1vhs1). RsbRB-only cells may still sense H_2_O_2_ but less sensitively; however, in our initial testing, the fast transition to lethality as H_2_O_2_ concentrations rise precluded our testing higher concentrations. RsbRB-only cells displayed a sharper and stronger response to acid stress than either wild-type or RsbRA-only cells (Fig. 5B; Movie S12 at https://doi.org/10.5061/dryad.2ngf1vhs1). The acid stress response was synchronous and transient, distinguishing it from the ethanol or NaCl response of RsbRB-only cells. These results suggest that the RsbRB protein differs from RsbRA in that RsbRB can elicit different σ^B^ response patterns according to the identity of the environmental stressor, at least among the tested stressors.

10.1128/mbio.02001-22.3 FIG S3 P*_rsbV_-*mNeonGreen (σ^B^ reporter) traces for individual RsbRB-only cell lineages in different stressors. Traces from individual cells, corresponding to the ensemble and average traces shown in Fig. 5, are shown. Each column shows traces from five individual cells challenged with the stressor shown at the top of the column. EtOH, 2% ethanol; H_2_O_2_, 0.005% hydrogen peroxide; NaCl, 500 mM NaCl; pH, shift from pH 6.5 to 6.25. Download FIG S3, JPG file, 3.0 MB. Copyright © 2022 Hamm et al. 2022 Hamm et al. https://creativecommons.org/licenses/by/4.0/ This content is distributed under the terms of the Creative Commons Attribution 4.0 International license.

### The RsbRC-only response is characterized by repetition and insensitivity to oxidative stress.

**FIG 6:**
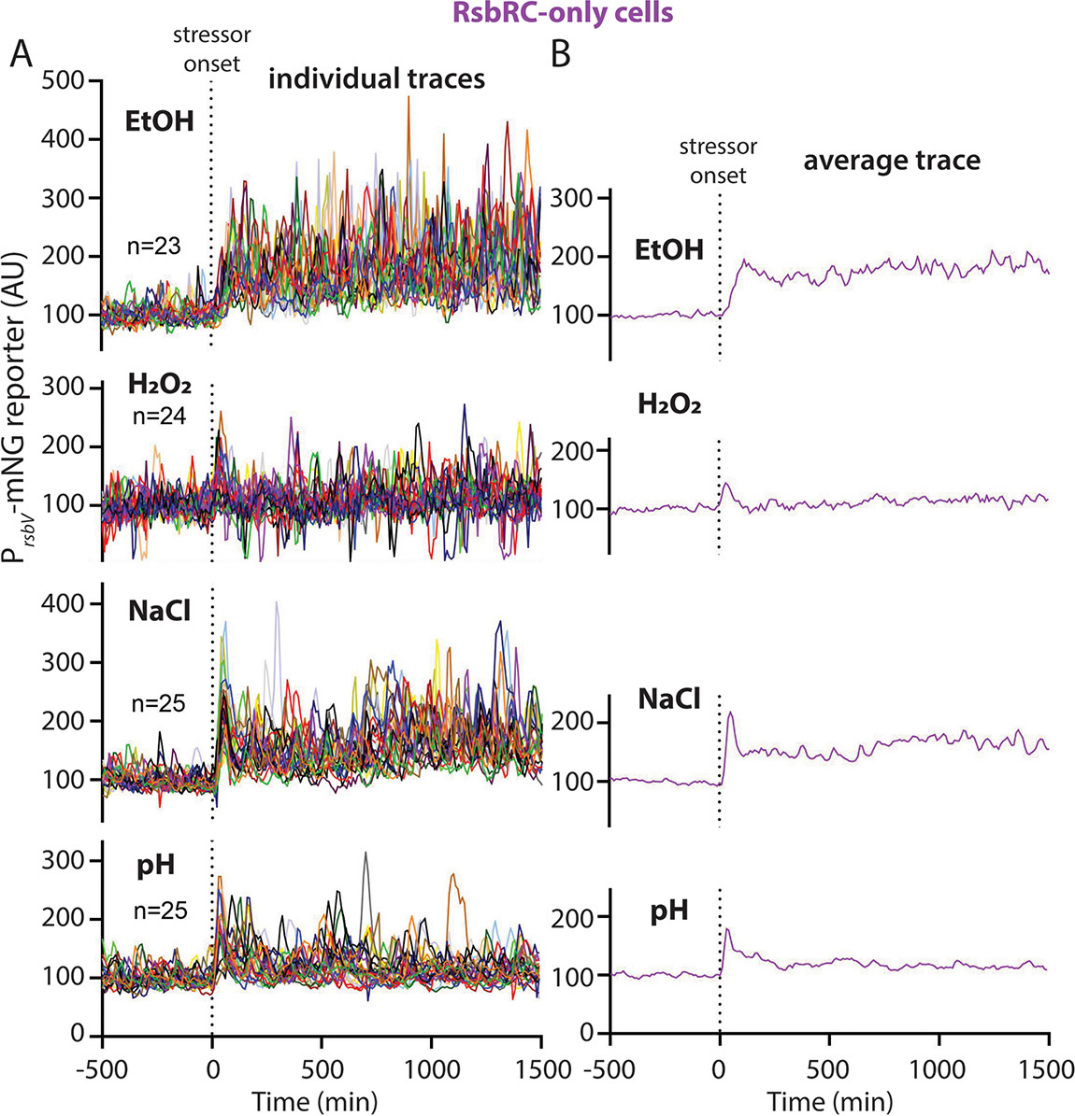
(A) Individual-lineage intensity traces of a stress-responsive P*_rsbV_*-mNeonGreen reporter in a an RsbRC-only strain (MTC1765) before and after the addition (indicated by vertical dotted line) of the indicated stressors. The stressors were 2% ethanol, 0.005% H_2_O_2_, 500 mM NaCl, and pH shift from 6.5 to 6.25. (B) Average intensity of the P*_rsbV_*-mNeonGreen reporter corresponding to the single-cell traces from panel A. Representative individual traces are shown in Fig. S4.

Having uncovered evidence that RsbRB-only cells can differentiate between stressors, we next tested RsbRC-only cells. RsbRC-only cells were originally characterized in ethanol as resembling RsbRB-only cells but with a greater response magnitude (Fig. 2B) (16). Namely, a sustained average response after stressor onset was composed at the single-cell level of stochastic, repeated σ^B^ activation events (Fig. 2A and 6A; Fig. S7; Movie S13 at https://doi.org/10.5061/dryad.2ngf1vhs1). We observed here that this pattern was largely preserved in other stressors; for example, NaCl and acid stress both resulted in repeated responses among single cells throughout the duration of stress exposure (Fig. 6A; Fig. S4 and S6; Movies S14 and S16 at https://doi.org/10.5061/dryad.2ngf1vhs1). However, both NaCl and acid stress elicited initial synchronous peaks, unlike the more gradual, asychronous response to ethanol (Fig. 6A). Overall, despite some similarities, RsbRC exhibited several differences from RsbRB across our panel of stressors. RsbRB-only cells respond more quickly and synchronously to ethanol than RsbRC, which takes longer to respond but then maintains a higher average response (Fig. 5 and 6). The patterns are reversed in NaCl stress, with which RsbRC-only cells show the faster and more synchronous response and RsbRB-only cells respond more slowly but mount a higher average sustained response (Fig. 5 and 6). Both RsbRC-only and RsbRB-only cells showed weak responses to oxidative stress, with RsbRC mediating a slightly stronger, transient response (Fig. 5 and 6). The patterns displayed by RsbRC-only cells strengthen the evidence that stressor identity can affect the σ^B^ responses mediated by different RsbR paralogs.

10.1128/mbio.02001-22.4 FIG S4 P*_rsbV_-*mNeonGreen (σ^B^ reporter) traces for individual RsbRC-only cell lineages in different stressors. Traces from individual cells, corresponding to the ensemble and average traces shown in Fig. 6, are shown. Each column shows traces from five individual cells challenged with the stressor shown at the top of the column. EtOH, 2% ethanol; H_2_O_2_, 0.005% hydrogen peroxide; NaCl, 500 mM NaCl; pH, shift from pH 6.5 to 6.25. Download FIG S4, JPG file, 2.0 MB. Copyright © 2022 Hamm et al. 2022 Hamm et al. https://creativecommons.org/licenses/by/4.0/ This content is distributed under the terms of the Creative Commons Attribution 4.0 International license.

10.1128/mbio.02001-22.7 FIG S7 Distributions of intervals between response peaks in selected strain/stressor combinations. To detect whether the repeated σ^B^ responses observed in certain strains had any characteristic frequency, a set of at least five randomly selected cells from two different strains (RsbRC-only, RsbRD-only) and two different stressors (EtOH and NaCl) were manually analyzed for inter-response peak times (between 40 and 62 intervals were analyzed for each strain/stressor combination). Thresholds of 150 and 200 arbitrary fluorescence units were used to identify peaks in NaCl and EtOH, respectively. The interpeak times were then plotted as histograms (20-min bins) to display the frequency distribution. Download FIG S7, TIF file, 2.1 MB. Copyright © 2022 Hamm et al. 2022 Hamm et al. https://creativecommons.org/licenses/by/4.0/ This content is distributed under the terms of the Creative Commons Attribution 4.0 International license.

### The RsbRD-only response largely resembles the RsbRC-only response.

**FIG 7:**
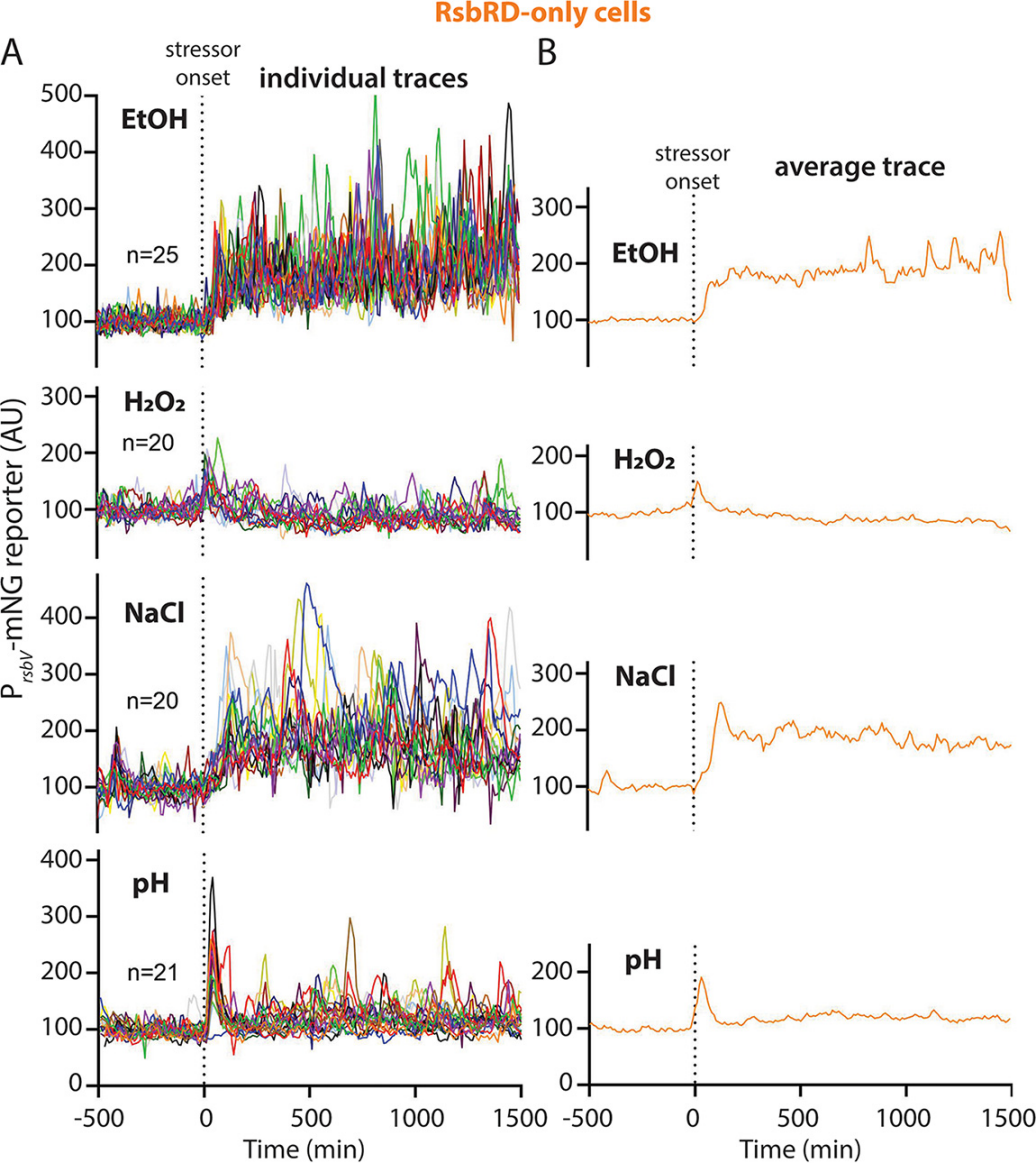
(A) Individual-lineage intensity traces of a stress-responsive P*_rsbV_*-mNeonGreen reporter in an RsbRD-only strain (MTC1767) before and after the addition (indicated by vertical dotted line) of the indicated stressors. The stressors were 2% ethanol, 0.005% H_2_O_2_, 500 mM NaCl, and pH shift from 6.5 to 6.25. (B) Average intensity of the P*_rsbV_*-mNeonGreen reporter corresponding to the single-cell traces from panel A. Representative individual traces are shown in Fig. S5.

Within wild-type cells containing all four RsbR paralogs, RsbRA, RsbRB, and RsbRC are expressed at similar levels during growth, while RsbRD is reportedly present at a much lower level (38). RsbRD levels rise only mildly during stressed conditions (38), leaving RsbRD as a minority player among the RsbR paralogs in the stressosome. In response to ethanol, cells containing only RsbRD looked similar to cells containing only RsbRC, but with a slightly weaker initial response that modestly increased in magnitude over the duration of exposure (Fig. 7A and 7B; Fig. S5 and S6; Movie S17 at https://doi.org/10.5061/dryad.2ngf1vhs1). Like RsbRC-only cells, RsbRD-only cells also showed a very weak, synchronous, and transient response to oxidative stress and a stronger but still synchronous and largely transient response to acid stress (Fig. 7A and 7B; Movies S18 and S20 at https://doi.org/10.5061/dryad.2ngf1vhs1). We observed a similar response to NaCl stress between RsbRD and RsbRC, with a stochastically repeated response following an initial synchronous response to the introduction of stress, although RsbRC-only cells exhibited a sharper and more synchronous initial peak (Fig. 6 and 7; Movie S19 at https://doi.org/10.5061/dryad.2ngf1vhs1). Our results show that RsbRD-only cells are similar to RsbRC-only cells in their response patterns and magnitude, and that the responses of both of these paralogs are distinct from those mediated by RsbRB. Collectively, our findings indicate that while the wild-type and RsbRA-only responses have a stereotypical response shape that varies only in magnitude among different stressors, the, RsbRB-, RsbRC-, and RsbRD-only responses appear to distinguish among different stressors in terms of their consequent σ^B^ response profiles.

10.1128/mbio.02001-22.5 FIG S5 P*_rsbV_-*mNeonGreen (σ^B^ reporter) traces for individual RsbRD-only cell lineages in different stressors. Traces from individual cells, corresponding to the ensemble and average traces shown in Fig. 7, are shown. Each column shows traces from five individual cells challenged with the stressor shown at the top of the column. EtOH, 2% ethanol; H_2_O_2_, 0.005% hydrogen peroxide; NaCl, 500 mM NaCl; pH, shift from pH 6.5 to 6.25. Download FIG S5, JPG file, 2.0 MB. Copyright © 2022 Hamm et al. 2022 Hamm et al. https://creativecommons.org/licenses/by/4.0/ This content is distributed under the terms of the Creative Commons Attribution 4.0 International license.

### The N-terminal region of RsbR paralogs influences response magnitude in hybrid fusions.

**FIG 8:**
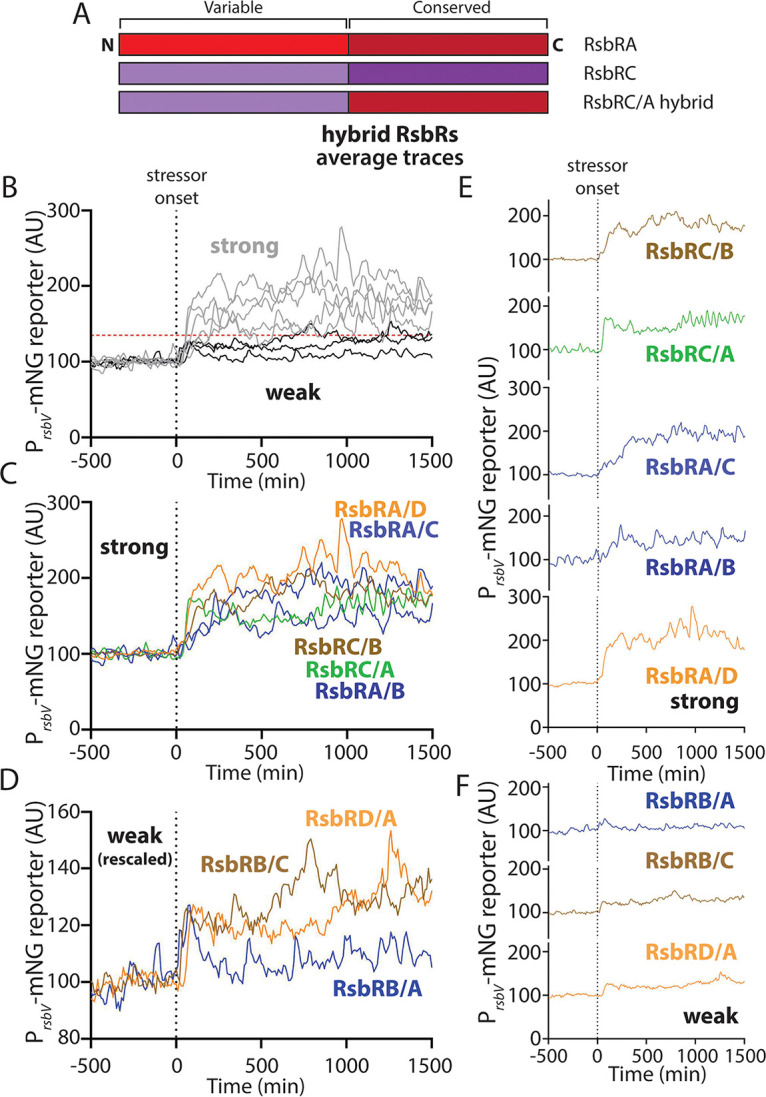
(A) Schematic illustrating the domain architecture of two different RsbR paralogs (RsbRA and RsbRC are shown as examples) and a hybrid between them, comprising the N-terminal variable domain or RsbRC and the C-terminal conserved domain of RsbRA. The schematic also illustrates the nomenclature of the example RsbRC/A hybrid. (B) Average traces of P*_rsbV_*-mNeonGreen reporter in hybrid strains (see strain table for complete genotypes and accession numbers) before and after addition (indicated by the vertical dotted line) of 2% ethanol. High average responses (gray lines) and low average responses (black) were distinguished, with the horizontal dotted line at 135 AU as a visual guide for “strong” and “weak” responses. (C) Individual average traces of P*_rsbV_*-mNeonGreen reporter in the indicated hybrid strains with a strong response (A or C in the N terminus) before and after addition of 2% ethanol. (D) Individual average traces of P*_rsbV_*-mNeonGreen reporter in the indicated hybrid strains with a weak response (B or D in the N terminus) before and after addition of 2% ethanol. (E) Traces of strong-response strains (as in panel B) displayed individually. (F) Traces of weak-response strains (as in panel C) displayed individually.

Except for RsbRA, we observed that each individual RsbR paralog showed σ^B^ response profiles that differed from paralog to paralog and from stressor to stressor. The RsbR paralogs are characterized by a largely conserved C-terminal domain and an N-terminal domain that varies among the paralogs (Fig. 1C and 8A). Therefore, we hypothesized that the observed differences among the RsbR paralogs in their response profiles is due to the differences in their primary sequences. As an initial test of this hypothesis, we investigated which region of RsbR is responsible for mediating the response profile. Our strategy was to engineer hybrid proteins composed of the variable region of one paralog and the conserved region of another. For example, a hybrid with the variable domain of RsbRC and the conserved region of RsbRA, joined at the STAS domain linker, was termed an RsbRC/A hybrid (Fig. 8A) (48). We inserted by markerless allelic replacement each hybrid-encoding gene at the locus of the C terminus (i.e., the *rsbRC/A* gene would be at the *rsbRA* locus) as the only source of RsbR in the cell. We constructed several hybrid combinations to learn whether any trends in response pattern were associated with particular variable or conserved sequences. For the sake of simplicity, we limited this initial analysis to 2% ethanol stress.

First, we observed that none of the hybrids perfectly mimicked the response of one of the constituent paralogs. Instead, nearly all the responses showed a repeated pattern of various magnitudes when challenged with ethanol. The response profiles of the eight hybrids we tested bifurcated into two rough classifications, with some initial responses being “weak” (below the dashed horizontal line in Fig. 8B) and others being “strong” (above the dashed horizontal line in Fig. 8B). We noted that the weak, sometimes barely detectable responses were observed when the hybrid contained the variable region of either RsbRB or RsbRD (Fig. 8D and 8F; Movies S24, S25, and S27 at https://doi.org/10.5061/dryad.2ngf1vhs1). Nonetheless, the RsbRB/C and RsbRD/A hybrids showed a weak but repeated response throughout the duration of stress exposure. Conversely, RsbRB/A was the only tested hybrid that showed a transient response, with a weak response peak (Fig. 8B, D, and 8F; Movie S27 at https://doi.org/10.5061/dryad.2ngf1vhs1). While the variable regions of RsbRB and RsbRD corresponded with weak responses, the variable regions of RsbRA and RsbRC were associated with stronger responses, all of which were sustained rather than transient (Fig. 8C and 8E; Movies S21 to S23, S26, and S28 at https://doi.org/10.5061/dryad.2ngf1vhs1). However, the speed of the response onset varied among the hybrids. The RsbRC/A and RsbRA/D hybrids both showed a fast onset (steep slope in Fig. 8C and 8E), whereas a slower onset was observed for RsbRC/B, RsbRA/C, and RsbRA/B (shallower slope in Fig. 8C and 8E). This pattern suggests that the RsbRA and RsbRD C-terminal regions mediate a faster onset, whereas the RsbRB and RsbRC C-terminal regions mediate a slower onset to ethanol stress. In sum, these data suggest that the downstream σ^B^ response profile mediated by a given RsbR protein is not solely dependent upon the variable N-terminal region traditionally thought to be the sensing domain. Instead, it appears that both halves of the protein contribute to the timing and magnitude of the stress response. RsbRA is an exemplar of this principle, as its characteristic strong, synchronous, and transient response to ethanol was not recapitulated in any of the RsbRA-containing hybrids we constructed, suggesting that both halves of RsbRA are needed for its typical response. Further testing is warranted to determine which specific residues or regions within the RsbR paralogs allow for sensing stress and govern the resulting response profiles, not only in ethanol but across different environmental stressors.

## DISCUSSION

Our findings in this work highlight differences among the RsbR paralogs with respect to their σ^B^ response profiles when challenged with different environmental stressors. We also confirm the utility of microfluidics-coupled fluorescence microscopy for distinguishing different stress-response patterns with single-cell resolution. Collectively, our results indicate that wild-type cells do not substantially change the *timing* of their σ^B^ responses according to stressor—the response is always transient across stressors—but responses do show different magnitudes. What is clear from our work is that different RsbR paralogs elicit responses to different stressors that can depart from the wild-type response to a given stressor and, additionally, differ among the paralogs. Taking the response to NaCl as an example, wild-type and RsbRA-only cells display a sharp, strong, and transient response, whereas RsbRB-only cells show a slower-onset but sustained response. RsbRC-only cells show an initial spike but then maintain a higher sustained response level, while RsbRD-only cells show a response that largely mimics the RsbRB response. In contrast, H_2_O_2_ always elicited a fast and transient response, but the magnitude of the response varied from virtually undetectable (RsbRB) to intermediate (RsbRC, RsbRD) to strong (wild type, RsbRA). Hence, when considered in isolation, different RsbR paralogs can elicit different σ^B^ response patterns to different stressors. While the available evidence points to these differences being due to the identity of the RsbR paralog(s) in the cell, we also acknowledge the possibility of alternative models. For instance, RsbRA, RsbRB, and RsbRC are produced in roughly equal amounts, and RsbRD is produced more weakly in wild-type cells (38). As we produced each paralog from its native locus, we assume that our results are not affected by differences in their actual cellular levels, but we cannot rule out the formal possibility that the observed σ^B^ response patterns may be affected by the abundance of a particular RsbR paralog. Similarly, although our previous work using ethanol revealed qualitatively similar response patterns for each RsbR paralog that varied only in amplitude according to stressor concentration (16), we did not test here the possibility that other stressors may elicit different σ^B^ response patterns according to stressor concentration.

An important implication of RsbR paralog- and stressor-specific σ^B^ response patterns is that there may be multiple proximate signals within cells that can lead to stressosome activation and RsbT release. Presumably, the different response patterns—strong or weak, transient or repeated—are a product of how a given stress stimulus interacts with components of the stressosome to govern the kinetics of RsbT release and recapture. The relative roles of different molecular players in this process remain to be determined. For instance, consider the contrast between a slower-onset, sustained response composed of repeated activation events in individual cells, such as that observed for RsbRB in NaCl (Fig. 5) or RsbRC in ethanol (Fig. 6), and the transient response of RsbRA to NaCl (Fig. 4). Do the kinetics of RsbR/RsbS dephosphorylation by RsbX differ among RsbR paralogs, and does this process make an important contribution to the overall response pattern? Do different stimuli differ in their ability to elicit RsbR/RsbS phosphorylation by RsbT and the subsequent RsbT release? What is responsible for the apparently stochastic (Fig. 2 and 5–7; Fig. S7) repetition of σ^B^ activation in many cases? Such stochastic activation resembles a pattern previously seen with energy (mycophenolic acid) stress and recapitulated with induced expression of constitutively active RsbTU (49). We never observed long durations of σ^B^ activity at the single-cell level. Might sustained release of RsbT in single-RsbR strains (i.e., other than RsbRA) result in stochastic pulsing? These remain key questions for a molecular-level understanding of stressosome activation.

Having examined the responses of each RsbR paralog in isolation, an important outstanding question is how different RsbR paralogs interact when simultaneously present in stressosomes. The close similarity between the σ^B^ responses of wild-type and RsbRA-only cells across multiple stressors support our earlier suggestion (16) that RsbRA dominates the wild-type response, enforcing a transient pattern. Because the stressosome governs the σ^B^ response pattern by sequestering and releasing RsbT, one hypothetical way that RsbRA could enforce a transient response is by recapturing the RsbT molecules released upon stress onset, but with RsbRA in a refractory state that is resistant to subsequent activation and RsbT release. Because there appears to be an approximately 10-fold excess of RsbR proteins over RsbT (38), all cellular RsbT could in principle be bound by RsbRA in stressosomes, even in the presence of other RsbR paralogs. If most of the initially released RsbT molecules were recaptured on refractory RsbRA proteins, subsequent σ^B^ responses would be suppressed, even if other RsbR paralogs were in a state that would normally release RsbT. Such a scenario would explain why the distinct σ^B^ responses of other RsbR paralogs are unmasked when analyzed in isolation (Fig. 5 to 7). We previously speculated that other RsbR paralogs might dominate in stressors other than ethanol (16), but our present work argues that RsbRA dominates across different types of stressors. An important test of this model will be to analyze cells with pairs of RsbR paralogs to learn whether the presence of RsbRA is sufficient to mask the distinctive responses of a second RsbR paralog. It will also be interesting to learn whether one non-RsbRA paralog can dominate over another. For example, would cells containing both RsbRC and RsbRB respond more strongly (like RsbRC) or more weakly (like RsbRB) to H_2_O_2_?

Our initial analyses using hybrid fusion proteins lead us to conclude that the characteristically transient σ^B^ responses of RsbRA are the exception, not the rule, among the RsbR paralogs. In nearly every fusion combination we tested, we observed responses that more closely resembled a sustained (repeated in single cells) response than a transient, RsbRA-type response. From these data, it appears that both the N- and C-terminal halves of RsbRA are important for achieving a transient response. The fusion results also suggested that both halves of an RsbR protein influence the resulting response profile, with the variable (N-terminal) regions of RsbRA and RsbRC associated with stronger responses and the conserved (C-terminal) regions of RsbRA and RsbRD associated with a faster response onset. While it is a possibility that different cellular levels of the hybrid proteins affect the σ^B^ response profile, the observation that the σ^B^ reporter remains off in the absence of stress argues that the hybrid proteins are stable enough to form stressosomes and sequester RsbT in the absence of stressors. The next frontier in this line of inquiry is to identify key residues or regions within the N- or C-terminal halves of different paralogs that are responsible for their distinct σ^B^ profiles.

How do the distinct responses of RsbRB, RsbRC, and RsbRD contribute to the overall wild-type response, given how similar the RsbRA-only responses are to the wild type? One possibility is that the other (non-RsbRA) paralogs make a minor contribution to the overall response; for instance, they may abet the wild-type response to acid stress, which was weaker in RsbRA-only cells than in the wild type but was relatively strong in RsbRB-, RsbRC-, and RsbRD-only cells. It is perhaps more likely that the different paralogs play more prominent roles in natural conditions, in contrast to the highly controlled conditions of the laboratory. We engineered strains to produce only single-RsbR paralogs to probe the response capabilities of each, and we thus far have considered individual stressors under exponential-phase growth conditions. In nature, stationary-phase or slow-growing cells likely encounter multiple stressors simultaneously, and perhaps under such conditions, each RsbR paralog has an important function in conditioning the σ^B^ response for optimal survival. A fuller understanding of the role of each RsbR paralog will require us to examine simultaneous and sequential stressor combinations and different growth phases. In any case, our results underscore the idea that the stressosome represents a flexible, robust stress-response system that can distinguish among stressors and produce many different response profiles.

## MATERIALS AND METHODS

### Strains and growth conditions.

The bacterial strains used in this study are listed in Table 1 and in the S1 Text. B. subtilis strains were routinely grown in LB Lennox broth (10 g/liter tryptone, 5 g/liter yeast extract, 5 g/liter NaCl) or on Lennox agar plates fortified with 1.5% Bacto agar at 37°C. When appropriate, antibiotics (MLS: 0.5 μg/mL erythromycin and 2.5 μg/mL lincomycin; 100 μg/mL carbenicillin) were added to select for markers. All strains used for microfluidic analysis contained the *hag*_A233V_ point mutation to render cells immotile, thereby preventing cell loss from side channels without interfering with motility regulation (42). Markerless replacement of *rsbR* genes with hybrid versions was performed using the pMiniMAD vector (a gift of Daniel Kearns) for allelic replacement. Details of strain construction are given in the Supplemental Information, Text S1.

10.1128/mbio.02001-22.9 TEXT S1 Detailed modes of strain and plasmid construction, strains, and primer sequences. Download Text S1, PDF file, 0.3 MB. Copyright © 2022 Hamm et al. 2022 Hamm et al. https://creativecommons.org/licenses/by/4.0/ This content is distributed under the terms of the Creative Commons Attribution 4.0 International license.

**TABLE 1:** Strains used in this study

Strain or plasmid	Relevant genotype or description	Source or reference
MTC1761	3610 *hag*_A233V_ Δ*ytvA* Δ*rsbRB* Δ*rsbRC* Δ*rsbRD amyE*::*DG364-*P*_hyperspank_-mNeptune* (Cm^R^) *ywrK*::*DG1730-P_rsbV_-mNeonGreen* (Spc^R^)	16
MTC1763	3610 *hag*_A233V_ Δ*ytvA* Δ*rsbRA* Δ*rsbRC* Δ*rsbRD amyE*::*DG364-P_hyperspank_-mNeptune* (Cm^R^) *ywrK*::*DG1730-P_rsbV_-mNeonGreen* (Spc^R^)	16
MTC1765	3610 *hag*_A233V_ Δ*ytvA* Δ*rsbRA* Δ*rsbRB* Δ*rsbRD amyE*::*DG364-P_hyperspank_-mNeptune* (Cm^R^) *ywrK*::*DG1730-P_rsbV_-mNeonGreen* (Spc^R^)	16
MTC1767	3610 *hag*_A233V_ Δ*ytvA* Δ*rsbRA* Δ*rsbRB* Δ*rsbRC amyE*::*DG364-P_hyperspank_-mNeptune* (Cm^R^) *ywrK*::*DG1730-P_rsbV_-mNeonGreen* (Spc^R^)	16
MTC1801	3610 *hag*_A233V_ Δ*ytvA amyE*::*DG364-P_hyperspank_-mNeptune* (Cm^R^) *ywrK*::*DG1730-P_rsbV_-mNeonGreen* (Spc^R^)	16
MTC2540	3610 *hag*_A223V_ Δ*ytvA* Δ*rsbRB* Δ*rsbRC* Δ*rsbRD rsbRA*::*rsbRC/A amyE*::*DG364-P_hyperspank_-mNeptune* (Cm^R^) *ywrK*::*DG1730-P_rsbV_-mNeonGreen* (Spc^R^); RsbRC/A hybrid as only source of RsbR within the cell	This study
MTC2541	3610 *hag*_A223V_ Δ*ytvA* Δ*rsbRA* Δ*rsbRB* Δ*rsbRD rsbRC*::*rsbRA/C amyE*::*DG364-P_hyperspank_-mNeptune* (Cm^R^) *ywrK*::*DG1730-P_rsbV_-mNeonGreen* (Spc^R^); RsbRA/C hybrid as only source of RsbR within the cell	This study
MTC2542	3610 *hag*_A223V_ Δ*ytvA* Δ*rsbRA* Δ*rsbRC* Δ*rsbRD rsbRB*::*rsbRC/B amyE*::*DG364-P_hyperspank_-mNeptune* (Cm^R^) *ywrK*::*DG1730-P_rsbV_-mNeonGreen* (Spc^R^); RsbRC/B hybrid as only source of RsbR within the cell	This study
MTC2543	3610 *hag*_A223V_ Δ*ytvA* Δ*rsbRA* Δ*rsbRB* Δ*rsbRD rsbRC*::*rsbRB/C amyE*::*DG364-P_hyperspank_-mNeptune* (Cm^R^) *ywrK*::*DG1730-P_rsbV_mNeonGreen* (Spc^R^); RsbRB/C hybrid as only source of RsbR within the cell	This study
MTC2544	3610 *hag*_A223V_ Δ*ytvA* Δ*rsbRB* Δ*rsbRC* Δ*rsbRD rsbRA*::*rsbRD/A amyE*::*DG364-P_hyperspank_-mNeptune* (Cm^R^) *ywrK*::*DG1730-P_rsbV_-mNeonGreen* (Spc^R^); RsbRD/A hybrid as only source of RsbR within the cell	This study
MTC2545	3610 *hag*_A223V_ Δ*ytvA* Δ*rsbRA* Δ*rsbRB* Δ*rsbRC rsbRD*::*rsbRA/D amyE*::*DG364-P_hyperspank_-mNeptune* (Cm^R^) *ywrK*::*DG1730-P_rsbV_-mNeonGreen* (Spc^R^); RsbRA/D hybrid as only source of RsbR within the cell	This study
MTC2546	3610 *hag*_A223V_ Δ*ytvA* Δ*rsbRB* Δ*rsbRC* Δ*rsbRD rsbRA*::*rsbRB/A amyE*::*DG364-P_hyperspank_-mNeptune* (Cm^R^) *ywrK*::*DG1730-P_rsbV_-mNeonGreen* (Spc^R^); RsbRB/A hybrid as only source of RsbR within the cell	This study
MTC2547	3610 *hag*_A223V_ Δ*ytvA* Δ*rsbRA* Δ*rsbRC* Δ*rsbRD rsbRB*::*rsbRA/B amyE*::*DG364-P_hyperspank_-mNeptune* (Cm^R^) *ywrK*::*DG1730-P_rsbV_-mNeonGreen* (Spc^R^); RsbRA/B hybrid as only source of RsbR within the cell	This study

### Microfluidic apparatus setup and media.

We used polydimethyl siloxane (PDMS) microfluidic devices. One version was dimensionally identical to that previously described (42), while a second was very similar to the previously described version (42), with shallow surrounding channels. A third version was dimensionally similar but lacked the shallow surrounding channels and had a shorter central cell-confining channel. We used this version when challenging cells with salt stress, as salt-treated cells tended to fill the shallow side channels (Fig. S8). We speculate that NaCl treatment may cause cells to contract slightly or become more flexible (50). The second and third versions were custom fabricated by ConScience AB (Mölndal, Sweden). Cured devices (10:1 Sylgard 184) cast on a silicon master (with SU-8 features) were punched with a 0.75-mm biopsy specimen punch to create holes to connect the fluidics using 21-gauge blunt needles. The devices were bonded to isopropyl alcohol-cleaned glass coverslips by oxygen-plasma treatment at ~200 mTorr O_2_ for 15 s at 30 W and baked at 65°C for at least 1 h before use. The devices were passivated with growth medium containing 1 mg/mL bovine serum albumin (BSA) before cell loading. The cells were grown in shaking culture to stationary phase (optical density at 600 nm [OD_600_] = ~4 to 5), filtered through a 5-μm filter to remove cell chains, concentrated by centrifugation at 5,000 × *g* for 10 min, and loaded into the device using gel-loading tips. The cells were then spun into the side channels of the device in a custom-designed microcentrifuge adaptor at 6,000 × *g* for 10 min. The fluidics were then connected to the device and run at 35 μL/min for approximately 20 min to flush out excess cells before being run at 1.5 μL/min for imaging. Imaging was not initiated until the cells in the device had resumed uniform exponential growth.

10.1128/mbio.02001-22.8 FIG S8 Microfluidic devices with and without a shallow channel surround. (A) Micrographs illustrating the filling of the shallow region surrounding the central cell channel with cells undergoing NaCl treatment. At an early time point (0 min), the cells were confined to the central deep channel. However, 200 min later, cells began to fill the shallow surrounding region, and the region was completely filled with cells 300 min later. (B) Schematic of cells confined in the central cell channel in a double-layer device (with two feature depths) with shallow surrounding region. The channel length is not to scale. (C) Schematic of cells confined in a single-layer device to prevent cell escape from a single-file line. The length is not to scale, but such channels are shorter to ensure diffusion of nutrients and additives to cells. Download FIG S8, TIF file, 2.8 MB. Copyright © 2022 Hamm et al. 2022 Hamm et al. https://creativecommons.org/licenses/by/4.0/ This content is distributed under the terms of the Creative Commons Attribution 4.0 International license.

The media used for fluidics always contained 0.1 mg/mL BSA as a passivation agent to limit cell adhesion to the device during flow. The fluidics were fed by 20-mL syringes in six-channel syringe pumps (New Era Pump Systems, Farmingdale, NY) that were connected by 21-gauge blunt needles to Tygon flexible tubing with an inner diameter (ID) of 0.02 in. To permit medium switches, two banks of syringes were used: one for the one for the prestress phase containing plain medium and the other for the stress phase containing the stressor. Each pair of syringes (minus and plus stressor) was joined with a polypropylene 1.6-mm-ID Y connector with 200-series barbs; 2-cm lengths of flexible silicone tubing (0.04-in. ID, 0.085-in. outer diameter) were used to connect the Tygon lines to the two input branches of the Y connector. The lengths of silicone tubing facilitated the placement of small binder clips to one or the other branch to make pinch valves. A 1-cm length of silicone tubing was used to connect a 10-cm length of Tygon tubing to the output of the Y connector. The output Tygon tubing was directly connected to a bent 21-gauge blunt needle that was then connected to the PDMS device, and a similarly constructed needle-tube combination was used to carry the outflow of the device to a waste beaker.

The environmental stressors used in this study included ethanol, salt, oxidative, and acid stress. We used lower concentrations of the stressors in the microfluidic platform than in previous reports testing the same stressors in culture flasks. We attribute this difference to the fact that in a closed (flask) culture, a defined and finite amount of stressor may be neutralized over time, whereas the microfluidic system is constantly replenished with respect to stressor. For ethanol stress, we added 2% EtOH to LB + 0.1 mg/mL BSA; the prestress medium was LB + 0.1 mg/mL BSA alone. For oxidative stress, we used 0.005% H_2_O_2_ added to LB + 0.1 mg/mL BSA. For acid stress, we used LB at a pH of 6.25 + 0.1 mg/mL BSA, and the prestress medium was LB at pH 6.5 + 0.1 mg/mL BSA. pH was adjusted by adding HCl or NaOH as needed to LB. Because LB Lennox medium already contains NaCl, we used a salt-free formulation of LB termed LBK because of its potassium phosphate (10 g/liter tryptone, 5 g/liter yeast extract, 21 mM K_2_HPO_4_, 11 mM KH_2_PO_4_) + 0.1 mg/mL BSA as the prestress medium and for salt stress added 500 mM NaCl to LBK + 0.1 mg/mL BSA.

Every strain and medium combination in this study was tested in at least two separate experiments (either completely separate lanes in a microfluidic device or separate microfluidic devices). If we suspected that reporter activity might be spurious (due to cell clogs or medium-flow stoppage), we repeated the experiment additional times to obtain consistent results. The lineages plotted in figures are representative and in some cases are taken from multiple experiments.

### Medium switching.

The experiments were always initiated in stressor-free medium, and this initial growth phase typically lasted approximately 10 to 12 h before the switch. In the initial phase, pinch valves were closed on the stressor-containing branch of the fluidics, and the corresponding syringe pump was paused. At the switch, the syringe pump with stressor-free medium was paused, the binder clips were carefully moved to the stressor-free branch of the Y connectors, and the other (stressor-containing) syringe pump was activated (at 2.5 μL/min). Tests with marker beads and dyes indicated that the second medium took approximately 50 min to reach the cells in the device. The switch apparatus was housed within a temperature-controlled microscope enclosure during imaging (see below).

### Automated imaging.

Imaging was performed with a Nikon Eclipse Ti inverted microscope equipped with a Photometrics Prime 95B sCMOS camera, a 100× Plan Apo oil objective (NA 1.45, Nikon), an automated stage (Nikon), a Lumencor SOLA SE II 365 Light Engine fluorescent illumination system, and an OKO temperature-controlled enclosure in which the temperature was maintained at approximately 37°C during imaging. Image acquisition was performed using NIS-Elements AR 5.11.03 64-bit. Filter cubes for GFP and mCherry were used to image mNeonGreen and mNeptune, respectively. mNeonGreen (used for σ^B^ reporters) was imaged at approximately 33% illumination power with 200-ms exposures, and mNeptune (used for cell visualization) was imaged with at approximately 33% power with 400-ms exposures. The images were captured at 10-min intervals. Phase-contrast images were occasionally also captured.

### Lineage tracking and curation.

Lineage tracking and curation of the average mNeonGreen average intensity in mother cells was used to generate σ^B^ reporter traces. Lineages were manually filtered to retain only lineages that were tracked for >150 continuous frames. Images of cells were then stabilized to eliminate any movement or shifts during the time lapse by using the ImageJ plugin “Template Matching and Slice Alignment.” Because spontaneous cell death events and other anomalies (e.g., overcrowding of side channels) were associated with spurious peaks in reporter intensity, the full filtered set of lineages was then manually curated to remove spurious events. The mean mNeonGreen fluorescence value was then determined for each lineage by drawing a region of interest (ROI) box slightly smaller than the size of a cell in ImageJ and using the ImageJ plugin “Time Series Analyzer.” The position of the ROI, which otherwise remained static at each time point, was manually reviewed and corrected when necessary to ensure that the ROI contained the mother cell of each lineage for the duration of the experiment. The mean pixel intensity in the mNeonGreen channel in the ROI of the cell at each 10-min interval was collected and then corrected by subtraction of the average background adjacent to the cell to eliminate any fluctuations during the time lapse. To facilitate comparison between strains, as well as to correct for variations in imaging parameters among experiments (such as exposure and illumination intensity), we normalized the prestress value to 100 AU. To do so, the average fluorescence value of each lineage prior to stressor onset (average of the first 50 frames) was obtained; 100 was divided by this average value to obtain a normalization constant. The normalization constant was then applied at every time point for that lineage. This process was independently repeated for each lineage. The curated and corrected lineage sets were used to plot average traces and overlaid single-lineage (single-cell) traces.

The mean GFP values for all tracked lineages, both the raw background-corrected values and the normalized values, are available in Excel spreadsheets on Dryad at https://doi.org/10.5061/dryad.2ngf1vhs1. All supplementary movies and their legends are available on Dryad at https://doi.org/10.5061/dryad.2ngf1vhs1.
