# Supplementary figures and images for: Bacillus subtilis Stressosome Sensor Protein Sequences Govern the Ability To Distinguish among Environmental Stressors and Elicit Different σB Response Profiles
Source: mBio. 2022 Nov 21;13(6):e02001-22. doi: 10.1128/mbio.02001-22 (PMC9765535; doi:10.1128/mbio.02001-22)

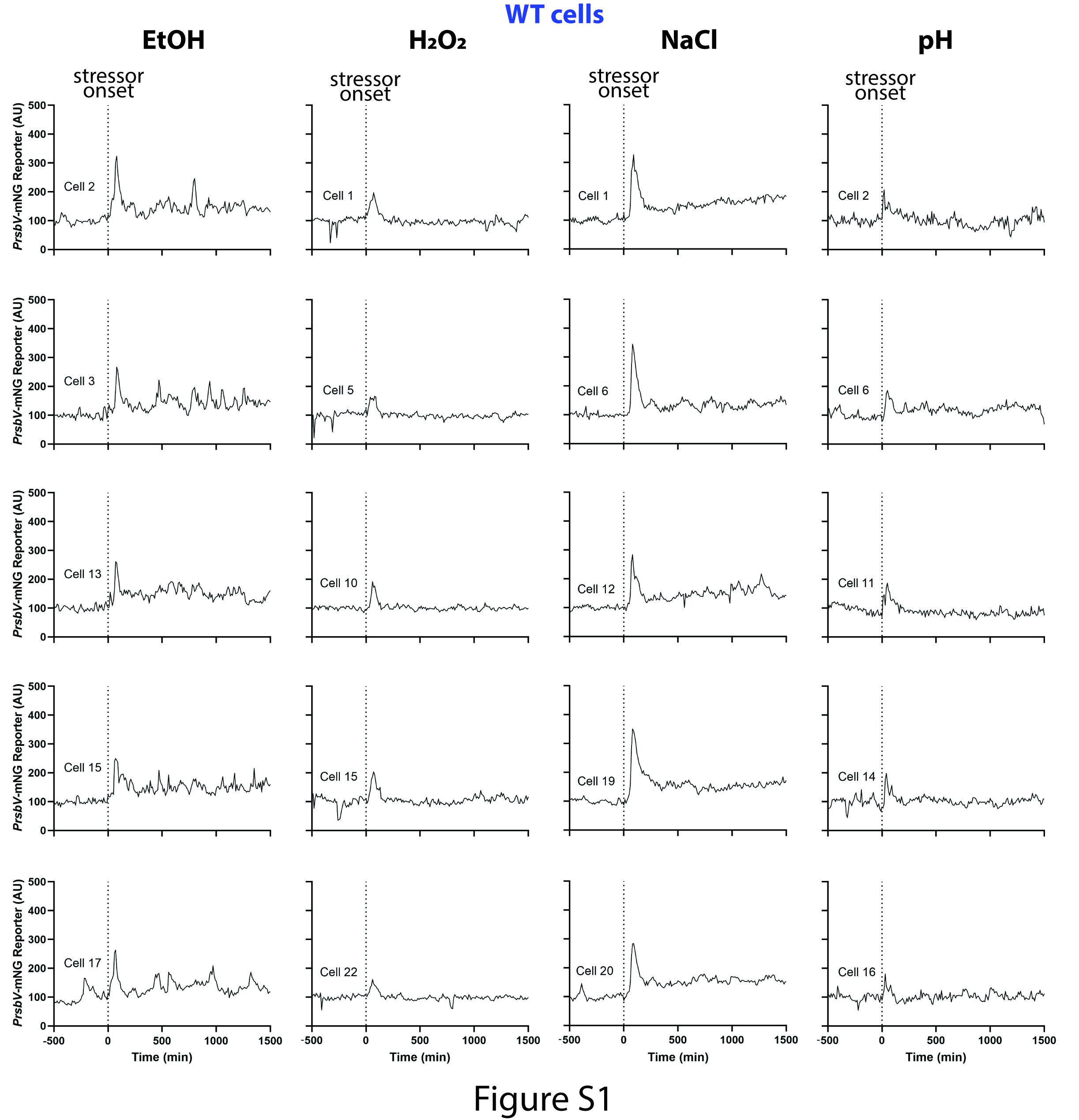

Supplement: FIG S1 [file mbio.02001-22-s0001.jpg]

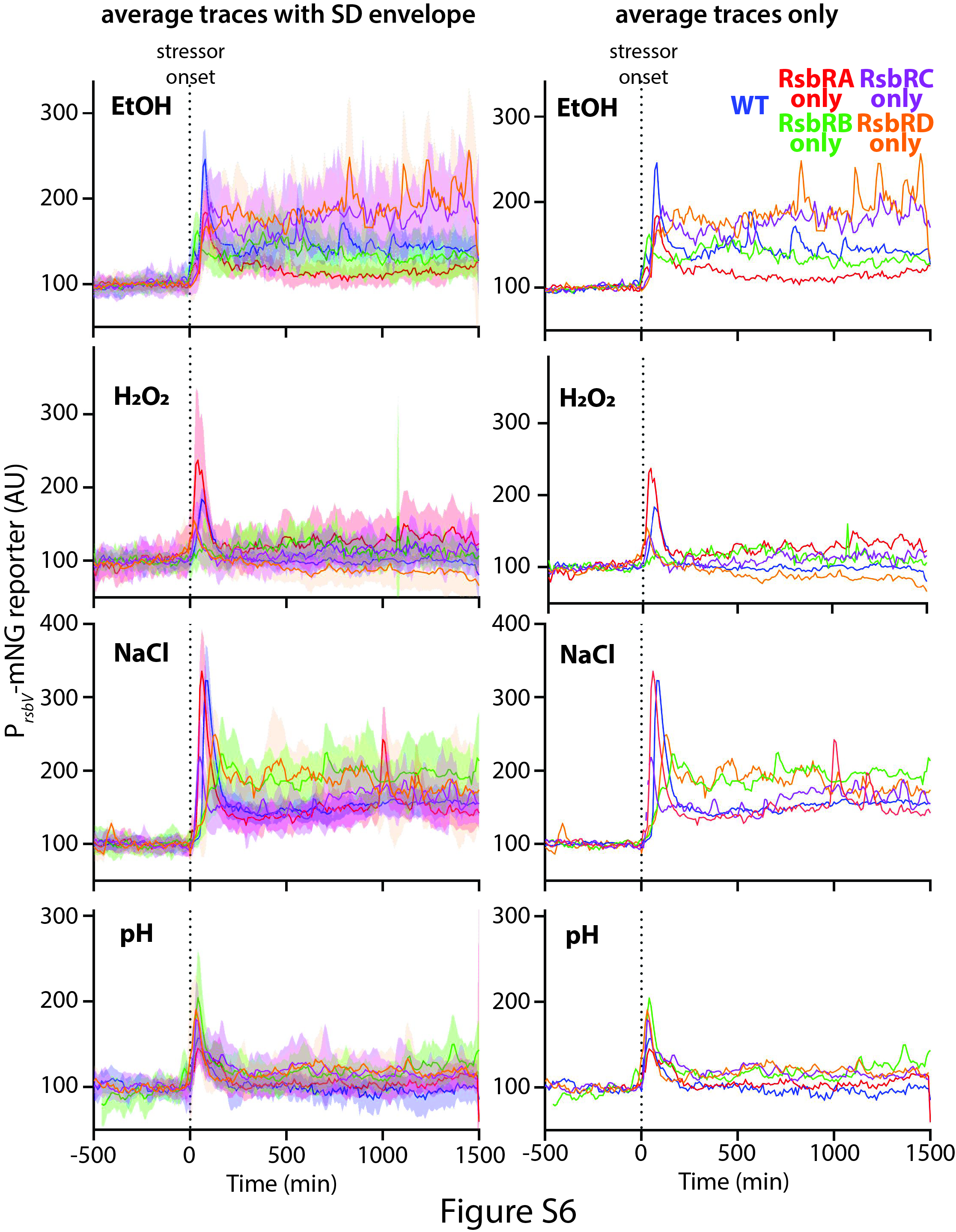

Supplement: FIG S6 [file mbio.02001-22-s0006.jpg]

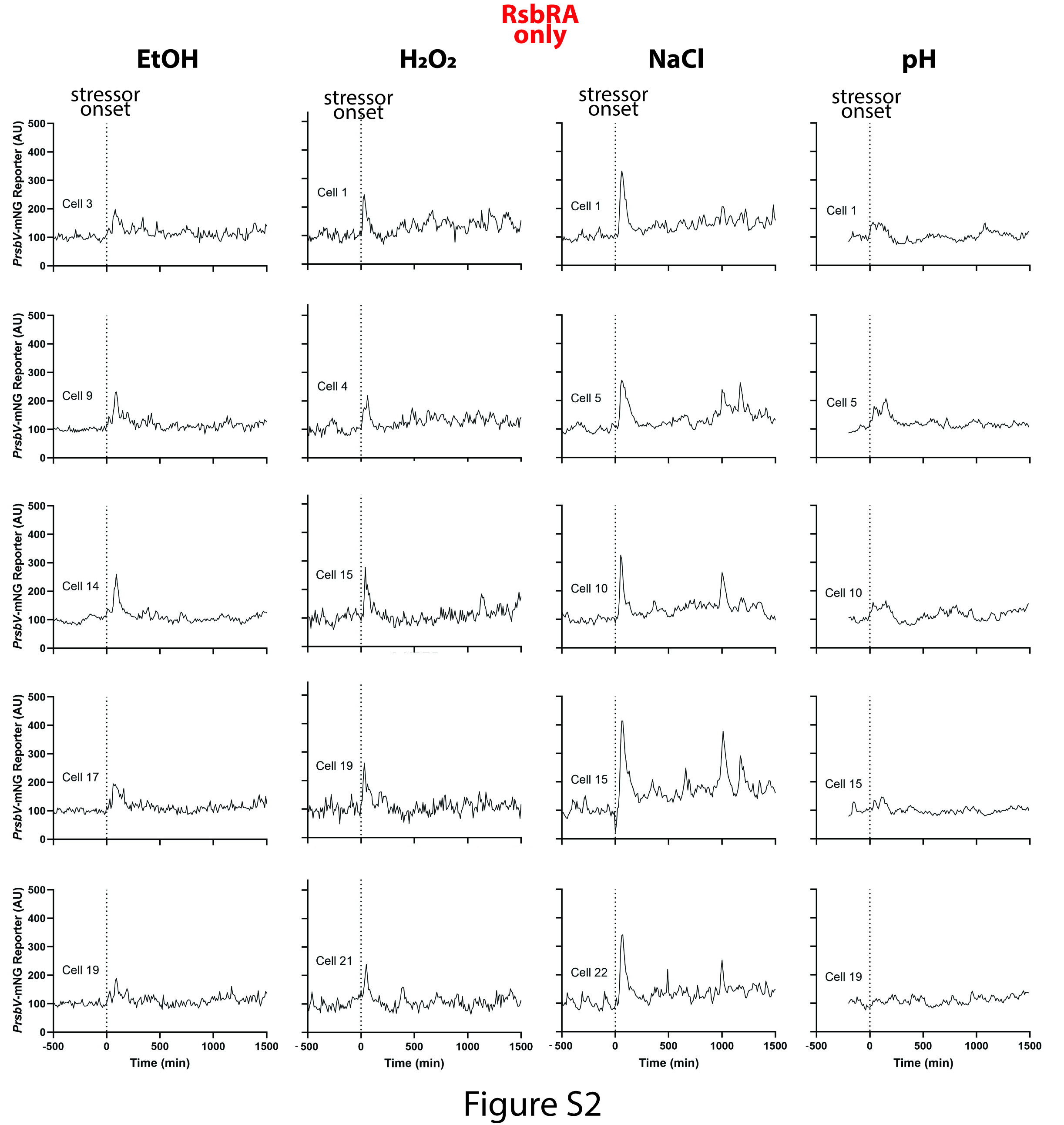

Supplement: FIG S2 [file mbio.02001-22-s0002.jpg]

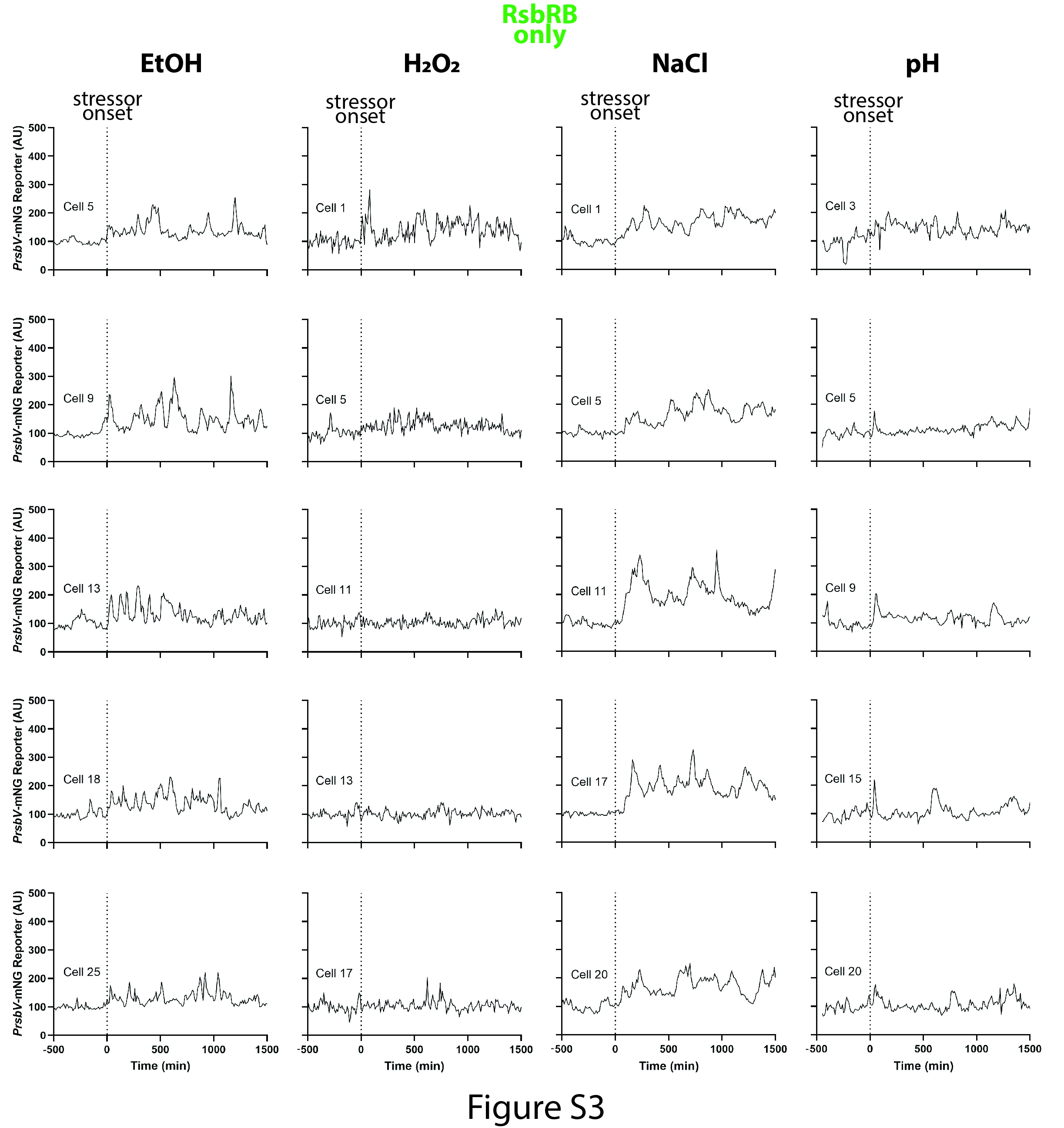

Supplement: FIG S3 [file mbio.02001-22-s0003.jpg]

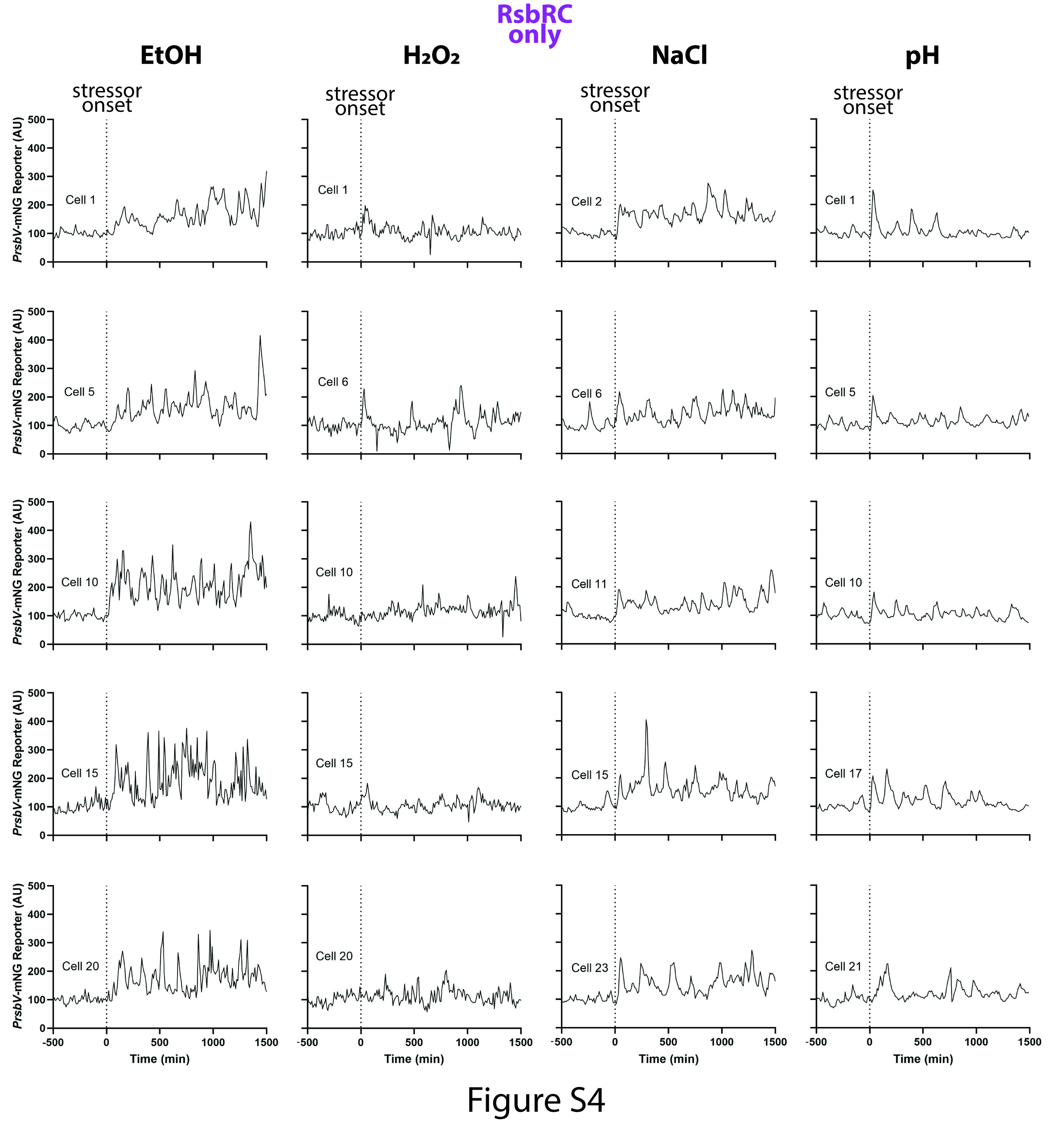

Supplement: FIG S4 [file mbio.02001-22-s0004.jpg]

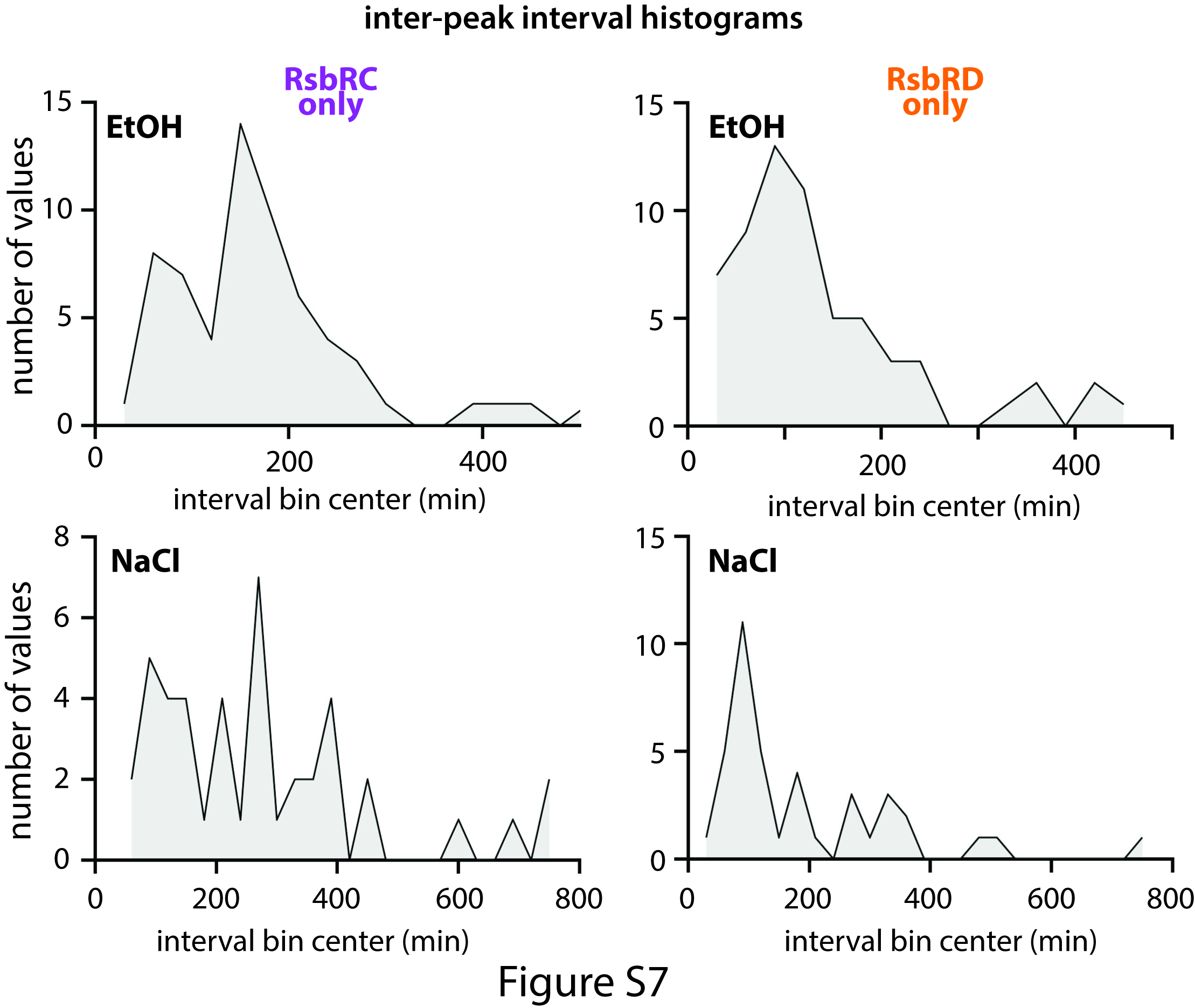

Supplement: FIG S7 [file mbio.02001-22-s0007.tif]

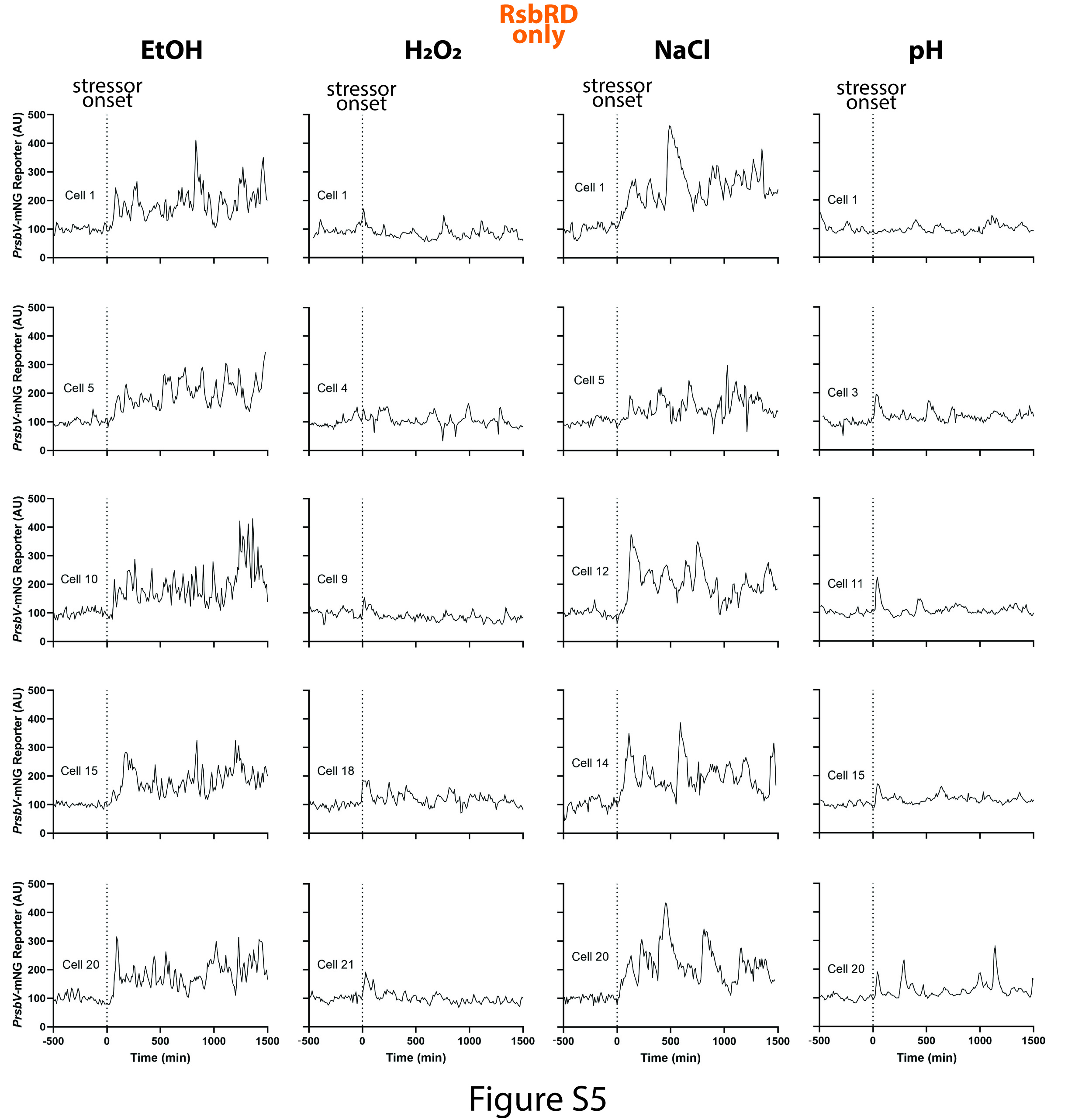

Supplement: FIG S5 [file mbio.02001-22-s0005.jpg]

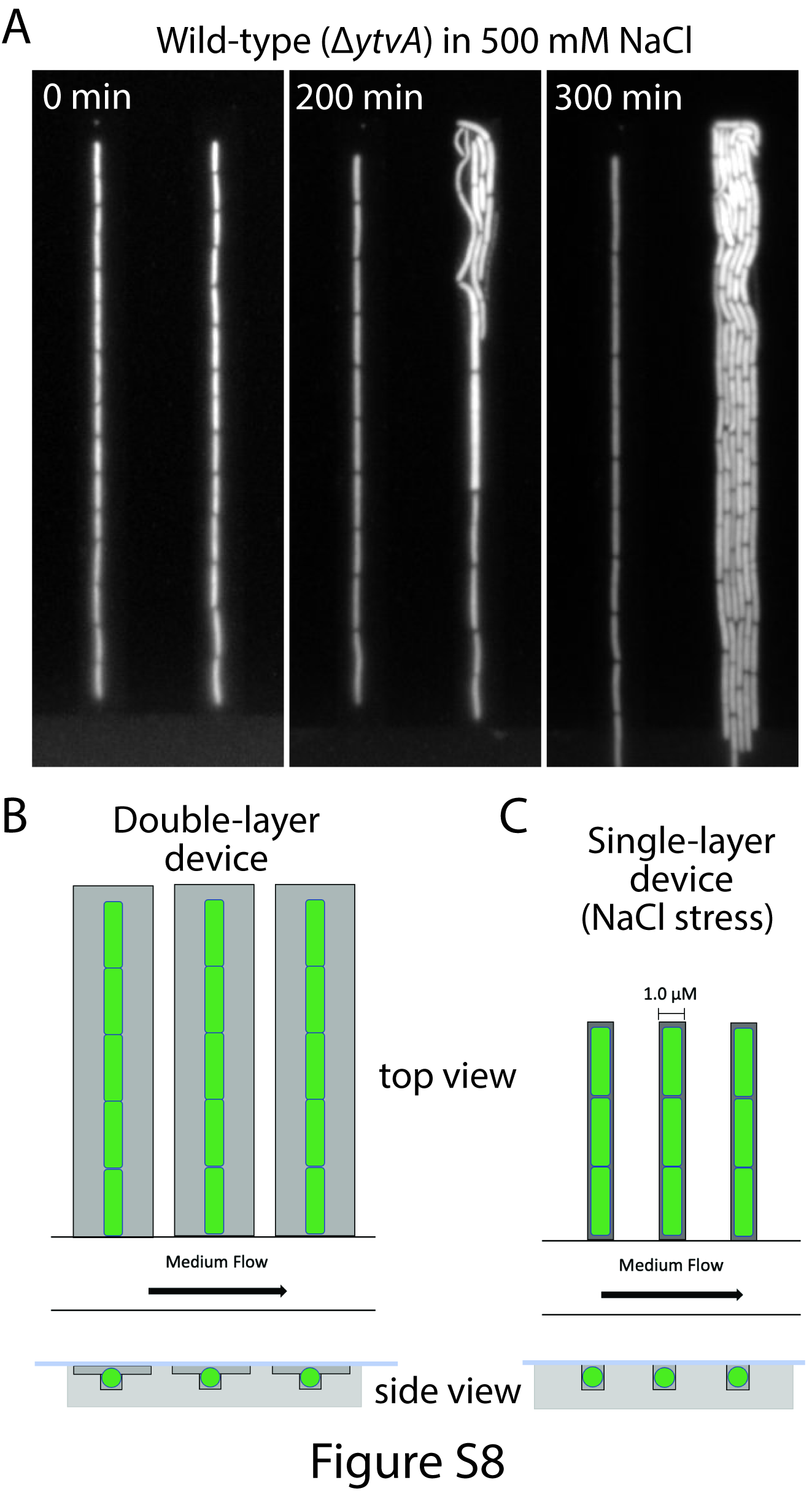

Supplement: FIG S8 [file mbio.02001-22-s0008.tif]
