# Supplementary material for: Bacillus subtilis Stressosome Sensor Protein Sequences Govern the Ability To Distinguish among Environmental Stressors and Elicit Different σB Response Profiles
Source: mBio. 2022 Nov 21;13(6):e02001-22. doi: 10.1128/mbio.02001-22 (PMC9765535; doi:10.1128/mbio.02001-22)
Supplement: TEXT S1 [file mbio.02001-22-s0009.pdf]

Supplemental Text S1. Detailed Modes of Strain and Plasmid Construction, Strains and Primer Sequences

The markerless gene replacement in the strains below were added in succession using the pminiMAD vector (gift of Daniel Kearns). A pminiMAD vector propagated in *E. coli* and containing the desired gene was directly transformed into PY79 via competence {Wilson, 1968 #1148} and selected on MLS (0.5 µg/ml erythromycin and 2.5 µg/ml lincomycin). A phage SPP1 lysate was prepared from that intermediate strain, and the recipient strain was phage-transduced with the PY79 strain containing the desired chromosomally integrated miniMAD vector and again selected on MLS. Five to 10 transductants were then inoculated into liquid LB and kept in exponential phase at approximately 25°C for several hours to permit plasmid excision before being repeatedly diluted and grown in liquid LB at 37°C (restrictive for plasmid replication) to promote loss of excised plasmid. The cells were then plated, and single colonies were screened for the successful replacement by colony PCR, restreaked for single colonies, patched on plain LB and LB/MLS plates to verify plasmid loss, restreaked, verified by PCR and stored at -80°C.

MTC2540

MTC 1761 was transduced with lysate from PY79 *pminiMAD-rsbRC/A* as described above to produce 3610 *hagA233V ΔytvA ΔrsbRB ΔrsbRC ΔrsbRD rsbRA::RsbRC/A*.

MTC2541

MTC 1765 was transduced with lysate from PY79 *pminiMAD-rsbRA/C* as described above to produce 3610 *hagA233V ΔytvA ΔrsbRA ΔrsbRB ΔrsbRD rsbRC::RsbRA/C*.

MTC2542

MTC 1763 was transduced with lysate from PY79 *pminiMAD-rsbRC/B* as described above to produce 3610 *hagA233V ΔytvA ΔrsbRA ΔrsbRC ΔrsbRD rsbRB::RsbRC/B*.

MTC2543

MTC 1765 was transduced with lysate from PY79 *pminiMAD-rsbRB/C* as described above to produce 3610 *hagA233V ΔytvA ΔrsbRA ΔrsbRB ΔrsbRD rsbRC::RsbRB/C*.

MTC2544

MTC 1761 was transduced with lysate from PY79 *pminiMAD-rsbRD/A* as described above to produce 3610 *hagA233V ΔytvA ΔrsbRB ΔrsbRC ΔrsbRD rsbRA::RsbRD/A*.

MTC2545

MTC 1767 was transduced with lysate from PY79 *pminiMAD-rsbRA/D* as described above to produce 3610 *hagA233V ΔytvA ΔrsbRA ΔrsbRB ΔrsbRC rsbRD::RsbRA/D*.

MTC2546

MTC 1761 was transduced with lysate from PY79 *pminiMAD-rsbRB/A* as described above to produce 3610 *hagA233V ΔytvA ΔrsbRB ΔrsbRC ΔrsbRD rsbRA::RsbRB/A*.

MTC2547

MTC 1763 was transduced with lysate from PY79 *pminiMAD-rsbRA/B* as described above to produce 3610 *hagA233V ΔytvA ΔrsbRA ΔrsbRC ΔrsbRD rsbRB::RsbRA/B*.

Plasmid construction

pminiMAD-rsbRC/A
Plasmid pminiMAD was linearized with EcoRI and HindIII. An upstream fragment containing the 5' flanking region upstream of *rsbRA* was amplified with primers 689/776. The middle fragment, containing the gene sequence encoding the variable region of RsbRC, was amplified with primers 777/778. The downstream fragment, containing the gene sequence encoding the conserved region of RsbRA and the flanking 3' region was amplified with primers 779/692. Each fragment contained an overlapping region with the adjacent fragment to allow stitching of all pieces together. All fragments were gel purified, and then isothermally assembled {Gibson, 2009 #989} with the linearized pminiMAD. The resulting plasmid was propagated in *E. coli* and confirmed by sequencing.

pminiMAD-rsbRA/C
Plasmid pminiMAD was linearized with EcoRI and HindIII. An upstream fragment containing the 5' flanking region upstream of *rsbRC* was amplified with primers 694/948. The middle fragment, containing the gene sequence encoding the variable region of RsbRA, was amplified with primers 949/950. The downstream fragment, containing the gene sequence encoding the conserved region of RsbRC and the flanking 3' region was amplified with primers 951/700. Each fragment contained an overlapping region with the adjacent fragment to allow stitching of all pieces together. All fragments were gel purified, and then isothermally assembled {Gibson, 2009 #989} with the linearized pminiMAD. The resulting plasmid was propagated in *E. coli* and confirmed by sequencing.

pminiMAD-rsbRA/D
Plasmid pminiMAD was linearized with EcoRI and HindIII. An upstream fragment containing the 5' flanking region upstream of *rsbRD* was amplified with primers 701/1058. The middle fragment, containing the gene sequence encoding the variable region of RsbRA, was amplified with primers 1059/1060. The downstream fragment, containing the gene sequence encoding the conserved region of RsbRD and the flanking 3' region was amplified with primers 1061/704. Each fragment contained an overlapping region with the adjacent fragment to allow stitching of all pieces together. All fragments were gel purified, and then isothermally assembled {Gibson, 2009 #989} with the linearized pminiMAD. The resulting plasmid was propagated in *E. coli* and confirmed by sequencing.

pminiMAD-rsbRD/A
Plasmid pminiMAD was linearized with EcoRI and HindIII. An upstream fragment containing the 5' flanking region upstream of *rsbRA* was amplified with primers 689/1070. The middle fragment, containing the gene sequence encoding the variable region of RsbRD, was amplified with primers 1071/1072. The downstream fragment, containing the gene sequence encoding the conserved region of RsbRA and the flanking 3' region was amplified with primers 1073/692. Each fragment contained an overlapping region with the adjacent fragment to allow stitching of all pieces together. All fragments were gel purified, and then isothermally assembled {Gibson, 2009 #989} with the linearized pminiMAD. The resulting plasmid was propagated in *E. coli* and confirmed by sequencing.

pminiMAD-rsbRB/A
Plasmid pminiMAD was linearized with EcoRI and HindIII. An upstream fragment containing the 5' flanking region upstream of *rsbRA* was amplified with primers 689/1062. The middle fragment, containing the gene sequence encoding the variable region of RsbRB, was amplified with primers 1063/1064. The downstream fragment, containing the gene sequence encoding the conserved region of

RsbRA and the flanking 3’ region was amplified with primers 1065/692. Each fragment contained an overlapping region with the adjacent fragment to allow stitching of all pieces together. All fragments were gel purified, and then isothermally assembled {Gibson, 2009 #989} with the linearized pminiMAD. The resulting plasmid was propagated in *E. coli* and confirmed by sequencing.

pminiMAD-rsbRA/B
Plasmid pminiMAD was linearized with EcoRI and HindIII. An upstream fragment containing the 5’ flanking region upstream of *rsbRB* was amplified with primers 693/1066. The middle fragment, containing the gene sequence encoding the variable region of RsbRA, was amplified with primers 1067/1068. The downstream fragment, containing the gene sequence encoding the conserved region of RsbRB and the flanking 3’ region was amplified with primers 1069/696. Each fragment contained an overlapping region with the adjacent fragment to allow stitching of all pieces together. All fragments were gel purified, and then isothermally assembled {Gibson, 2009 #989} with the linearized pminiMAD. The resulting plasmid was propagated in *E. coli* and confirmed by sequencing.

pminiMAD-rsbRB/C
Plasmid pminiMAD was linearized with EcoRI and HindIII. An upstream fragment containing the 5’ flanking region upstream of *rsbRC* was amplified with primers 697/952. The middle fragment, containing the gene sequence encoding the variable region of RsbRB, was amplified with primers 953/954. The downstream fragment, containing the gene sequence encoding the conserved region of RsbRC and the flanking 3’ region was amplified with primers 955/700. Each fragment contained an overlapping region with the adjacent fragment to allow stitching of all pieces together. All fragments were gel purified, and then isothermally assembled {Gibson, 2009 #989} with the linearized pminiMAD. The resulting plasmid was propagated in *E. coli* and confirmed by sequencing.

pminiMAD-rsbRC/B
Plasmid pminiMAD was linearized with EcoRI and HindIII. An upstream fragment containing the 5’ flanking region upstream of *rsbRB* was amplified with primers 693/956. The middle fragment, containing the gene sequence encoding the variable region of RsbRC, was amplified with primers 957/958. The downstream fragment, containing the gene sequence encoding the conserved region of RsbRB and the flanking 3’ region was amplified with primers 959/696. Each fragment contained an overlapping region with the adjacent fragment to allow stitching of all pieces together. All fragments were gel purified, and then isothermally assembled {Gibson, 2009 #989} with the linearized pminiMAD. The resulting plasmid was propagated in *E. coli* and confirmed by sequencing.

**Strains or plasmid**	**Relevant genotype or description**	**Source or reference**
***E. coli* strain**		
MTC2037	NEB Turbo / pminiMAD-rsbRA/C	This study
***B. subtilis* strains**		
MTC52	PY79	Cabeen et al. 2017

MTC53	3610	Cabeen et al. 2017
HAM33	PY79 pminiMAD-rsbRA/C	This Study
HAM14	PY79 pminiMAD-rsbRC/B	This Study
HAM27	PY79 pminiMAD-rsbRB/C	This Study
HAM42	PY79 pminiMAD-rsbRD/A	This Study
HAM40	PY79 pminiMAD-rsbRA/D	This Study
HAM45	PY79 pminiMAD-rsbRB/A	This Study
HAM47	PY79 pminiMAD-rsbRA/B	This Study
**Plasmids**		
pminiMAD	Suicide plasmid for *B. subtilis* markerless allelic replacement	Lab Strain from Daniel Kearns
pminiMAD -RsbRC/A	pminiMAD-based markerless deletion plasmid for *rsbRC/A*	This Study
pminiMAD -RsbRA/C	pminiMAD-based markerless deletion plasmid for *rsbRA/C*	This Study
pminiMAD -RsbRC/B	pminiMAD-based markerless deletion plasmid for *rsbRC/B*	This Study
pminiMAD -RsbRB/A	pminiMAD-based markerless deletion plasmid for *rsbRB/C*	This Study
pminiMAD -RsbRD/A	pminiMAD-based markerless deletion plasmid for *rsbRD/A*	This Study
pminiMAD -RsbRA/D	pminiMAD-based markerless deletion plasmid for *rsbRA/D*	This Study
pminiMAD -RsbRB/A	pminiMAD-based markerless deletion plasmid for *rsbRB/A*	This Study
pminiMAD -RsbRA/B	pminiMAD-based markerless deletion plasmid for *rsbRA/B*	This Study
**Primer Name**	**Primer Sequence (often contains 5' extensions for assembly that do not anneal to the template)**	
689	AACAGCTATGACCATGATTACGCCAAGCTTCGGCTATATGGAAATGGCG	Cabeen et al. 2017
692	CGTTGTAAAACGACGGCCAGTGAATTCTTCCTCTGTCTGCGACCTG	Cabeen et al. 2017

693	AACAGCTATGACCATGATTACGCCAAGCTTTCGCCGCCAAGAACCTTC	Cabeen et al. 2017
696	CGTTGTAAAACGACGGCCAGTGAATTCTGTCGGCATCTCTCTCGGG	Cabeen et al. 2017
697	AACAGCTATGACCATGATTACGCCAAGCTTGGCAGCCATGAATTTTGCG	Cabeen et al. 2017
700	CGTTGTAAAACGACGGCCAGTGAATTCCAAGAGCTCATCAACGCTTGC	Cabeen et al. 2017
701	AACAGCTATGACCATGATTACGCCAAGCTTATATCCAAGCTGCACGTC	Cabeen et al. 2017
704	CGTTGTAAAACGACGGCCAGTGAATTCGTGCTGTTTTCCATGCTGAC	Cabeen et al. 2017
776	TTTTTGCCATCAAATTCGCTTACCTCCCAATAAAAAAC	This Study
777	AGCGAATTTGATGGCAAAAAACAAAAAATTATTCGAG	This Study
778	GCGCAGACAGTTCAGTAATCATATCTTTTTGGGCC	This Study
779	GATTACTGAACTGTCTGCGCCGCTTATCC	This Study
948	AGTCTGGTTCGACATGATTGATCACCTCTTTTAAA	This Study
949	AAGAGGTGATCAATCATGTCGAACCAGACTGTATA	This Study
950	GGAGCGCTCAACTCTTGCAGCG	This Study
951	GCTGCAAGAGTTGAGCGCTCCGGTC	This Study
952	TTCATTCAGTTTCATGATTGATCACCTCTT	This Study
953	CATTTAAAAGAGGTGATCAATCATGAAACTGAATGA	This Study
954	GCACGATGACCGGAGCGCTCAATTCCAATATCAT	This Study
955	TGATATTGGAATTGAGCGCTCCGG	This Study
956	TTTTTTGTTTTTTGCCATGACACTGCTCCT	This Study
957	GAGCAGTGTCATGGCAAAAAACAA	This Study
958	GGGTAATGACAGGTGAGCTCAATTCAGTAA	This Study

959	ATATGATTACTGAATTGAGCTCACCTGTCATTA=	This Study
1058	ACAGTCTGGTTCGACATCTTAATGAGTTACC	This Study
1059	AACTCATTAAGATGTCGAACCAGACTGTAT	This Study
1060	GCATAATCGGCGCACTCAACTCTTGCAG	This Study
1061	AGAGTTGAGTGCGCCGATTATGC	This Study
1062	TCAGTTTCATCAAATTCGCTTACCTCCCAAT	This Study
1063	GGAGGTAAGCGAATTTGATGAAACTGAATGA	This Study
1064	CGGCGCAGACAGTAATTCCAATATCATTT	This Study
1065	TGATATTGGAATTACTGTCTGCGCCGCTTA	This Study
1066	GTTCGACATGACACTGCTCCTTTCCCCAAC	This Study
1067	GGAAAGGAGCAGTGTCATGTCGAAC	This Study
1068	CAGGTGAGCTTAACTCTTGCAGCGCGATTT	This Study
1069	GCGCTGCAAGAGTTAAGCTCACCTGTCATT	This Study
1070	ATCAAGAGCTATCATCAAATTCGCTTACC	This Study
1071	AAGCGAATTTGATGATAGCTCTTGATCAG	This Study
1072	AGCGGCGCAGACAGTTCATTAATCATTTC	This Study
1073	AATGATTAATGAACTGTCTGCGCCGCTTA	This Study
